# Positional Isomerism Influences pH-Responsive Release Kinetics in Methacryloyloxybenzoic Acid Polymeric Prodrugs

**DOI:** 10.3390/polym18212789

**Published:** 2026-11-08

**Authors:** Hebrón Vázquez-Estudillo, Giovanni Palomino-Vizcaino, Brisa G. Hernández-Ramírez, Lilia A. Hurtado-Ayala, Jonathan Vincent Lopez-Baena, Teresa G. Rodriguez-Tellez, Iván Cordova-Guerrero, Lucia E. Azuara-Alvarez, Arturo Estolano-Cobián, Kenia Palomino-Vizcaino, José M. Cornejo-Bravo, Héctor Magaña

**Affiliations:** 1 Faculty of Chemical Sciences and Engineering, Autonomous University of Baja California, University Boulevard No. 14418, Otay Mesa, Tijuana 22390, Mexico; hebron.vazquez@uabc.edu.mx (H.V.-E.); brisa.hernandez@uabc.edu.mx (B.G.H.-R.); lilyhurtado@uabc.edu.mx (L.A.H.-A.); vlopez16@uabc.edu.mx (J.V.L.-B.); rodriguezt1@uabc.edu.mx (T.G.R.-T.); icordova@uabc.edu.mx (I.C.-G.); estolano.arturo@uabc.edu.mx (A.E.-C.); kenia.palomino@uabc.edu.mx (K.P.-V.); jmcornejo@uabc.edu.mx (J.M.C.-B.); 2 Faculty of Health Sciences, Autonomous University of Baja California, 1000 University Boulevard, Valle de Las Palmas, Tijuana 22260, Mexico; gpalomino@uabc.edu.mx (G.P.-V.); lucia.azuara@uabc.edu.mx (L.E.A.-A.)

**Keywords:** polymeric prodrugs, pH-responsive polymers, positional isomerism, hydrolytic susceptibility, controlled release, methacryloyloxybenzoic acid, planktonic antibacterial biomaterials, salicylic acid delivery

## Abstract

Polymeric prodrugs capable of pH-responsive drug release are promising platforms for localized and controlled drug delivery. While positional isomerism is known to affect the physicochemical properties of MBA polymers, a comparative evaluation of its effect on drug release kinetics and biological activity is lacking. In this study, three methacryloyloxybenzoic acid (MBA) polymeric prodrugs derived from *ortho*-, *meta*-, and *para*-hydroxybenzoic acid (HBA) isomers were synthesized to comparatively evaluate the influence of substituent position on HBA release profiles and biological performance. The *o*-, *m*-, and *p*-MBA monomers and their corresponding polymers were synthesized and structurally characterized by FTIR-ATR, ^1^H-NMR and UV-Vis spectroscopy. HBA release profiles investigated under physiologically relevant pH conditions revealed markedly distinct release profiles among the polymers. Poly(*o*-MBA) exhibited rapid and near-complete release at intestinal and physiological pH, poly(*p*-MBA) showed sustained and intermediate release, and poly(*m*-MBA) displayed minimal release under all tested conditions, indicating that substituent position strongly influences HBA release, likely through differences in ester accessibility and hydrolytic susceptibility. The polymers also exhibited planktonic antibacterial activity against *Escherichia coli*, *Staphylococcus aureus*, and *Pseudomonas aeruginosa*, while BALB/3T3 fibroblast viability remained above 70% at a concentration of 0.5 mg/mL. These findings indicate that positional isomerism is a key structural determinant of pH-responsive release kinetics in MBA polymers and highlight their potential as versatile platforms for pharmaceutical and biomedical applications, particularly in the design of localized and controlled drug delivery systems.

## 1. Introduction

Localized bacterial infections and inflammation remain significant clinical challenges in various biomedical contexts [1,2,3]. To address these issues and reduce the risk of associated complications, Polymeric Drug Delivery Systems (PDDS) have been widely explored as platforms designed to achieve sustained and controlled release of bioactive agents in order to improve therapeutic performance [4]. Among these strategies, Polymeric Prodrugs (PP) are particularly noteworthy. In these systems, the active molecule is covalently integrated into the polymer backbone through stimuli-responsive linkages, such as ester, amide, or disulfide bonds, in an initially inactive form. These prodrugs become pharmacologically active only upon exposure to specific triggers, including pH variations, enzymatic activity, or other physicochemical stimuli [5,6,7], making them highly attractive for targeted and localized therapies.

Salicylic acid, or *o*-hydroxybenzoic acid (*o*-HBA), is a well-documented nonsteroidal anti-inflammatory drug (NSAID) that acts by inhibiting the COX-1 enzyme, reducing prostaglandin formation [8,9]. Beyond its anti-inflammatory activity, *o*-HBA and related phenolic acids (PAs), such as *m*-HBA and *p*-HBA, exhibit broad antimicrobial properties through multiple mechanisms, including the disruption of bacterial membranes, increased permeability, enzyme inhibition, and metabolic interference [10].

Licea et al. previously reported the synthesis of poly(*o*-, *m*-, and *p*-methacryloyloxybenzoic acid) (MBA polymers) via free-radical polymerization. In their study, they demonstrated that positional isomerism strongly influences polymer physicochemical properties, including acidity, chain rigidity, hydrogen-bonding interactions, hydrophobicity, and pH-responsive behavior [11]. Consistent with these observations, Magaña et al. demonstrated that poly(*o*-MBA) grafted onto polypropylene films behaved as a polymeric prodrug capable of sustained and pH-dependent salicylic acid release through hydrolysable ester bonds, highlighting that water accessibility and polymer–water interactions critically regulate ester bond hydrolysis [12].

While these studies establish that positional isomerism dictates the physicochemical properties of MBA polymers [11] and that poly(*o*-MBA) can function as a stimuli-responsive prodrug [12], a systematic comparison of the release kinetics, planktonic antibacterial activity, and cell viability across all three isomeric MBA PPs has not yet been reported. Building upon the evidence that positional isomerism modulates polymer rigidity, ionization, and polymer–water interactions [11,12], we hypothesized that the specific position of the aromatic substituent would critically govern not only the hydrolytic susceptibility and release profiles of the HBA isomers but also their resulting biological performance.

Therefore, the present study aimed to synthesize and characterize *o*-, *m*-, and *p*-MBA monomers and their corresponding MBA polymers, designed as PPs in which the hydroxybenzoic acid (HBA)-derived moiety is covalently attached to the polymer backbone through a hydrolysable ester linkage [11,12]. The methacrylate double bond of each MBA monomer enables free-radical polymerization, while the HBA-derived moiety remains attached to the resulting polymer as a pendant group. Hydrolysis of the ester linkage is expected to release the corresponding parent HBA [12,13]. The resulting materials were comparatively evaluated to determine the effect of substituent position on their pH-dependent HBA release profiles. Their planktonic antibacterial activity against clinically relevant pathogens and their effects on BALB/3T3 mouse fibroblast viability were also assessed. Although preventing biofilm formation is an ultimate goal for implant-associated applications, evaluating the intrinsic planktonic antibacterial activity of novel materials represents a necessary first step in characterizing their antimicrobial properties. Accordingly, the biological evaluation in this study initially focused on assessing the inhibition of planktonic bacterial growth by MBA polymers in suspension to establish their baseline antibacterial activity. Overall, this work investigates how positional isomerism influences HBA release kinetics and the biological properties of MBA-based polymeric prodrugs, providing a comparative assessment of their pH-responsive release profile, planktonic antibacterial activity, and effects on fibroblast viability.

## 2. Materials and Methods

### 2.1. Materials

For the synthesis of methacryloyloxybenzoic acid monomers (MBA monomers) and poly(*o*-, *m*-, and *p*-methacryloyloxybenzoic acid) (MBA polymers), *o*-hydroxybenzoic acid (*o*-HBA, salicylic acid, ≥99%), *m*-hydroxybenzoic acid (*m*-HBA, 97%), and *p*-hydroxybenzoic acid (*p*-HBA, 99%), as well as methacrylic anhydride (MAAH, 94%), 4-dimethylaminopyridine (DMAP, ≥99%), triethylamine (Et_3_N, ≥99%), 2,2′-azobis(2-methylpropionitrile) (AIBN, 98%), anhydrous *p*-dioxane (99.8%), anhydrous dichloromethane (CH_2_Cl_2_, ≥99.8%), magnesium sulfate (MgSO_4_, anhydrous, ≥99.5%), hydrochloric acid (HCl, 37%), deuterated chloroform (CDCl_3_, 99.8 atom % D, containing 0.03% *v*/*v* TMS), sodium 1-heptanesulfonate (≥98.0%), HPLC-grade acetonitrile, HPLC-grade methanol, glacial acetic acid, formic acid, and anhydrous toluene were purchased from Sigma-Aldrich (St. Louis, MO, USA) and used as received. Silica gel (200–400 mesh), petroleum ether, and diethyl ether used for column chromatography purification were also obtained from Sigma-Aldrich (St. Louis, MO, USA). Biological materials included BALB/3T3 murine embryonic fibroblasts (ATCC^®^ CCL-163™, Manassas, VA, USA) and bacterial reference strains *Staphylococcus aureus* (ATCC^®^ 29213™), *Escherichia coli* (ATCC^®^ 25922™), and *Pseudomonas aeruginosa* (ATCC^®^ 27853™). Cell culture reagents, Dulbecco’s Modified Eagle Medium (DMEM), Fetal Bovine Serum (FBS), L-glutamine, penicillin/streptomycin, dimethyl sulfoxide (DMSO), and phosphate-buffered saline (PBS), as well as Brain Heart Infusion (BHI) broth were supplied by Sigma-Aldrich (St. Louis, MO, USA). Cell viability was quantified using Cell Proliferation Kit I (MTT; Roche Diagnostics GmbH, Mannheim, Germany).

### 2.2. Synthesis and Polymerization of MBA Monomers

MBA monomers and MBA polymers were synthesized following established protocols [11,12]. Briefly, 6.91 g (50 mmol) of the corresponding hydroxybenzoic acid isomer (*o*-, *m*-, or *p*-HBA) and 0.49 g (4 mmol) of 4-dimethylaminopyridine (DMAP) were dissolved in a mixture of 8.42 mL (60 mmol) of triethylamine (Et_3_N) and 20 mL of anhydrous dichloromethane (CH_2_Cl_2_) under an inert atmosphere. The reaction mixture was cooled to 0 °C, and 7.50 g (50 mmol) of methacrylic anhydride (MAAH) was added dropwise under continuous stirring. The solution was stirred at 0 °C for 5 h and then allowed to react overnight (16 h) at room temperature. The organic reaction mixture was partitioned against 3 N HCL, dried over anhydrous MgSO_4_, filtered, and concentrated in vacuum. The resulting crude product was purified by silica gel column chromatography (200–400 mesh, eluent: petroleum ether/diethyl ether, 70:30 *v*/*v*). The isolated crystalline white solids were dried under vacuum for 24 h to yield pure *o*-MBA, *m*-MBA, and *p*-MBA monomers (Figure S1).

Poly(*o*-, *m*-, and *p*-MBA) were synthesized by free-radical polymerization using 2,2′-azobis(isobutyronitrile) (AIBN) as the thermal initiator. Briefly, 0.50 g (2.43 mmol) of the MBA monomer (*o*-MBA, *m*-MBA, and *p*-MBA) and AIBN (4.25 × 10^−3^ g, 0.02588 mmol; approximately 1.07 mol% relative to the monomer) were charged into a polymerization Schlenk flask. Dry *p*-dioxane (3.0 mL) was added to achieve a total initial monomer concentration of 0.81 M, and the mixture was then stirred at 0 °C under a nitrogen atmosphere until complete dissolution. The reaction mixture was degassed at −40 °C via five freeze-pump-thaw cycles on a vacuum line, backfilled with oxygen-free nitrogen, and hermetically sealed. After cooling to room temperature, the crude polymer solution was poured into a 20-fold excess of cold petroleum ether to precipitate the polymer. Purification was performed by dissolving the recovered solid in a minimal volume of dry *p*-dioxane and re-precipitating into petroleum ether; this process was repeated five times to ensure complete removal of unreacted monomer and residual initiator. Finally, the purified polymer was dried under reduced pressure (26 mbar) at 120 °C for 8 h [11].

MBA monomers and polymer yields were calculated gravimetrically as follows:

$$\mathrm{Yield}\;(\%)=\left(\frac{m_{DryProduct}}{m_{HBAorMonomerFeed}}\right)\times 100$$

where m_DryProduct_ is the mass of the dry, purified monomer or polymer recovered after esterification or polymerization (*g*) and m_HBAorMonomerFeed _is the initial mass of the HBA or MBA monomer (*g*). Reported yields represent the average of three independent synthesis batches (*n* = 3).

### 2.3. Characterization of HBA Isomers, MBA Monomers, and Polymers

#### 2.3.1. ^1^H-NMR and FTIR-ATR Analysis

^1^H-NMR spectra of MBA monomers were acquired using a Bruker Avance III HD 400 MHz spectrometer (Bruker BioSpin, Billerica, MA, USA), with CDCl_3_ as the solvent. For each measurement, 25 mg of the monomer was dissolved in 0.6 mL of CDCl_3_ and transferred to an NMR tube. Spectra were recorded at 303.1 K and processed using TopSpin software (version 5.0.0; Bruker BioSpin, Billerica, MA, USA). The chemical shifts are expressed as δ ppm values using TMS as the internal standard.

FTIR-ATR spectra of the solid HBA isomers, MBA monomers, and polymers were acquired using a Nicolet iS5 spectrometer equipped with an attenuated total reflectance (ATR) accessory (Thermo Fisher Scientific, Madison, WI, USA). Spectra were recorded over the wavenumber range of 4000–700 cm^−1^, with 16 scans collected per sample. The resulting spectra were processed and analyzed using OriginPro software (v2024; OriginLab Corporation, Northampton, MA, USA).

#### 2.3.2. UV Spectroscopy Characterization

The maximum absorption wavelengths of the HBA isomers, MBA monomers, and polymers were determined by UV–Vis spectroscopy using a Hach DR 6000 UV-VIS Spectrophotometer (Hach Company, Loveland, CO, USA). All samples were initially prepared in ethanol at a concentration of 1 mg/mL and subsequently diluted to 0.01 mg/mL (1:20) prior to analysis. Spectra were recorded over the range of 190–340 nm. Ethanol was used as a blank in matched quartz cuvettes to account for solvent absorption, particularly near its UV cutoff (approximately 205 nm).

#### 2.3.3. Determination of Apparent Polymer Molecular Weight by Static Light Scattering (SLS)

The apparent molecular weight (*M_W_*) of each MBA polymer was determined by Static Light Scattering (SLS) using a Zetasizer Nano ZS (Malvern Instruments Ltd., Malvern, UK) equipped with a He-Ne laser = 633 nm at a scattering angle of 173°. Measurements were performed at 25 °C, following a previously reported method [14]. The Debye plot was obtained with MBA polymer samples dissolved in anhydrous methanol at concentrations of 0.3, 0.7, and 1 mg/mL and filtered through 0.45 μm poly(tetrafluoroethylene) (PTFE) syringe filters prior to analysis. Toluene was used as the reference standard for the Rayleigh ratio, as its absolute scattering intensity is well-documented and universally accepted for instrument calibration [15]. In the calculation, the scattering intensity of the polymer solutions was measured relative to the toluene standard to determine the sample’s excess Rayleigh ratio, which is the fundamental variable required to construct the Debye plot and solve for the apparent *M_W_*. The solvent refractive index for anhydrous methanol was set to 1.328, and a refractive-index increment (dn/dc) of 0.14 mL/g was applied. Each concentration was measured in triplicate to ensure reproducibility.

### 2.4. MBA Polymer Biological Characterization

#### 2.4.1. Cell Viability Assay

The cytotoxic effects of MBA polymers and HBA isomers were evaluated in the presence of BALB/3T3 murine embryonic fibroblasts (ATCC^®^ CCL-163^TM^, Manassas, VA, USA). BALB/3T3 cell line was cultured in DMEM, 10% *v*/*v* FBS, 1% L-glutamate, and 1% penicillin/streptomycin at 37 °C in a humidified atmosphere containing 5% CO_2_. Cells were grown to approximately 85% confluence and detached from the culture flask. Cell morphology and distribution were examined using an inverted microscope before testing.

The BALB/3T3 cells were seeded at a density of 5 × 10^3^ cells/well in a 96-well culture plate and incubated for 24 h at 37 °C in a humidified atmosphere with 5% CO_2_. The culture medium was removed, and the BALB/3T3 cells were washed with sterilized PBS. Stock solutions of the MBA polymers and HBA isomers were prepared by first dissolving the compounds in DMSO, followed by dilution with DMEM to obtain a final concentration of 2 mg/mL in DMEM containing 2% DMSO (*v*/*v*). The resulting stock solutions were subsequently subjected to serial dilution with DMEM, filtered, and added to each well (100 µL/well) to achieve final concentrations of 0.125, 0.25, 0.5, and 1 mg/mL, corresponding to final DMSO concentrations of 0.125%, 0.25%, 0.5%, and 1% (*v*/*v*), respectively. Thus, the DMSO concentration was proportionally reduced with decreasing MBA polymer or HBA isomer concentration, with a maximum of 1% (*v*/*v*) DMSO at the highest concentration tested (1 mg/mL), as previously reported [13]. The BALB/3T3 cell line was incubated with the polymer/isomer dilutions for 24 h, and the metabolic activity was evaluated using a Cell Proliferation Kit I (MTT; Roche Diagnostics GmbH, Mannheim, Germany), according to the manufacturer’s instructions. Thereafter, the absorbance of the MTT reaction was recorded in triplicate at 620 nm with a Multiskan FC microplate reader (Thermo Fisher Scientific, Waltham, MA, USA). The results are expressed as cell viability percentages with respect to the control (1% DMSO).

#### 2.4.2. Bacterial Inhibition Assay

Bacterial inhibition assays for MBA polymers were carried out in the Microbiological Analysis Laboratory (FCQI, UABC) using 96-well plates and brain heart infusion (BHI) medium. MBA polymer samples were sterilized by UV irradiation. Stock solutions of the MBA polymers were prepared by first dissolving the compounds in DMSO, followed by dilution with BHI medium to obtain a final concentration of 2 mg/mL in BHI medium containing 2% DMSO (*v*/*v*). The resulting stock solutions were subsequently subjected to serial dilution with BHI medium and filtered to achieve final concentrations of 1, 0.5, 0.25, and 0.125 mg/mL, corresponding to DMSO concentrations of 1%, 0.5%, 0.25%, and 0.125% (*v*/*v*), respectively. Serial dilutions were prepared to proportionally reduce the DMSO concentration alongside the polymer concentration, with a maximum of 1% (*v*/*v*) DMSO at the highest concentration tested (1 mg/mL). A bacterial suspension equivalent to 0.5 McFarland (~1.5 × 10^8^ CFU/mL) was prepared for *Staphylococcus aureus* (ATCC^®^ 29213^TM^), *Escherichia coli* (ATCC^®^ 25922^TM^), and *Pseudomonas aeruginosa* (ATCC^®^ 27853^TM^). Polymer dilutions and the standardized inoculum were added to the 96-well plates and incubated at 37 °C in a controlled-environment incubator. After 24 h, absorbance was measured at 620 nm using a Thermo Scientific Multiskan microplate reader (Thermo Fisher Scientific, Waltham, MA, USA). To exclude any potential optical contributions from the polymer dispersions, polymer-only blanks (BHI medium containing the respective polymer concentrations and 1% *v*/*v* DMSO, without bacterial inoculum) were evaluated in parallel. The background absorbance of these blanks at 620 nm was systematically subtracted from the test samples prior to calculating the percentage of bacterial inhibition. Bacterial inhibition (%) was calculated by considering the untreated control (1% DMSO) as 0% inhibition.

#### 2.4.3. Statistical Analysis

Analysis of variance (ANOVA) and Tukey’s post hoc test were used to determine statistical significance in cell and bacterial studies. The results were obtained with a minimum of 3 replicates for each condition and are expressed as mean ± standard deviation (SD). Statistical analyses were performed using GraphPad Prism software (v10.0; GraphPad Software, San Diego, CA, USA).

### 2.5. HBA Isomers Release Profiles

To investigate the release profiles of the HBA isomers from the MBA polymers, solid dosage forms were prepared. The dried MBA polymers were ground, and 50 ± 0.2 mg of each polymer was placed between circular stainless-steel plates and compressed using a Carver press (Carver, Inc., Wabash, IN, USA). An applied load of 3000 kgf (equivalent to approximately 29.4 kN of compression force) was applied for 1 min, corresponding to a calculated compaction pressure of 221.7 MPa for a 13 mm diameter die. Each solid dosage form was individually placed inside a dialysis membrane (12–14 kDa MWCO; total surface area, 6,400 mm^2^) containing 5 mL of the corresponding release medium (donor compartment): an acidic NaCl solution at pH 1.2 or phosphate-buffered saline (PBS) at pH 6.8 or 7.4. The dialysis bags were then immersed in 45 mL of the same release medium, which served as the receiver medium. The systems were incubated at 37 °C in a VWR controlled-environment incubator shaker (VWR International, West Chester, PA, USA) under continuous agitation at 40 rpm, as previously reported [16]. At predetermined time points over 48 h, 1 mL aliquots were withdrawn from the receiver medium and immediately replaced with 1 mL of fresh release medium to maintain a constant receiver volume. The concentration of the released *o*-, *m*-, and *p*-HBA isomers was quantified by High-Performance Liquid Chromatography (HPLC) using calibration curves generated from standard solutions of the corresponding HBA isomers prepared at pH 1.2, 6.8, and 7.4. All experiments were performed in triplicate. The cumulative amount of HBA released was mathematically corrected for the replacement of the sampled medium at each time point.

The cumulative release calculations were adjusted to account for volume replacement at each time point. It should be noted that each 50 mg polymer tablet contains a maximum theoretical drug equivalent payload of approximately 34 mg. Because the donor compartment inside the dialysis membrane is limited to a 5 mL volume, the released drug could temporarily reach local saturation before diffusing into the 45 mL receiver medium, especially at low pH. Consequently, strict sink conditions cannot be guaranteed. Instead, this setup was designed to provide a reliable comparative assessment of release kinetics among the three isomers under standardized experimental conditions.

#### 2.5.1. Chromatographic Quantification of HBA Isomers

The HPLC system for chromatographic analysis consisted of an UltiMate™ 3000 HPLC Chromatograph (Thermo Fisher Scientific, Germering, Germany), equipped with a C18 column (250 mm × 4.6 mm, 5 µm, Hypersil GOLD). To ensure reliability, the chromatographic conditions were adopted directly from a standardized compendial method established in the Pharmacopoeia of the United Mexican States [17].

The mobile phase was prepared by dissolving 2 g of sodium 1-heptanesulfonate in a water/acetonitrile mixture (850:150, *v*/*v*), then adjusting the pH to 3.4 with glacial acetic acid. All mobile phases were filtered through a 0.22 µm nylon membrane and degassed prior to use. The separation was performed under isocratic elution for 6 min.

Samples were collected from the release media at 15, 30, 45, and 60 min, as well as 1.5, 2, 2.5, 3, 3.5, 4, 8, 12, 24, and 48 h at pH 1.2, 6.8, and 7.4, and 700 µL of each sample was diluted with 300 µL of a mixture of acetonitrile and formic acid (97:3). The samples were filtered through a 13 mm Acrodisc^®^ nylon syringe filter (Pall Corporation, Port Washington, NY, USA) with a 0.45 µm pore size. A 1 mL aliquot of the filtered sample was transferred into a 2 mL Thermo Scientific™ TTR-T vial (Rockwood, TN, USA) for injection into the HPLC system. The solvent flow rate was 1.2 mL/min, the injection volume was 10 µL, and the column temperature was maintained at 45 °C. The Photodiode Array Detector (PDA) wavelengths were set at 280, 288, and 245 nm for the *o*-, *m*-, and *p*-hydroxybenzoic acids (HBA isomers), respectively.

Qualitative analysis was performed based on retention times and absorption spectrum scanning using the Photodiode Array Detector (PDA). Quantitative analysis was conducted by calculating the Area Under the Curve (AUC) of the corresponding analytical peaks in the obtained chromatograms. Matrix-matched calibration curves were constructed in triplicate for each simulated dissolution medium (pH 1.2, 6.8, and 7.4). The corresponding calibration curves are presented in the Supplementary Materials (Figures S4–S6). The slope, intercept, coefficient of determination (*R^2^*), response factor (RF), standard deviation of the RF, and relative standard deviation (%RSD) were determined for each calibration curve. The %RSD was calculated according to the applicable equations established in the Pharmacopoeia of the United Mexican States [17]. The analytical parameters obtained for each isomer and dissolution medium are summarized in Table S2. The results were obtained with a minimum of 3 replicates for each condition and are expressed as mean ± standard deviation (SD). Statistical analyses were performed using GraphPad Prism software (v10.0; San Diego, CA, USA). The resulting data were processed using OriginPro software (v2024; OriginLab Corporation, Northampton, MA, USA).

To elucidate the release kinetics of the MBA polymers from solid dosage forms, cumulative release data obtained under each pH condition were fitted to mathematical kinetic models, including Zero-order, First-order, Higuchi, and Korsmeyer–Peppas equations, using the DDSolver add-in for Microsoft Excel [18]. Model fitting was restricted to data points within the fractional release interval *M_t/_M*_∞_ ≤ 0.60 (up to 60% cumulative release) to focus the analysis on the initial portion of the release profiles. Kinetic parameters (*k*_0_ in % · min^−1^; *k*_1_ in min^−1^; *k_H_* in % · min^−0.5^; *k_KP_* in % · min^−^*^n^*) and the adjusted coefficient of determination (*R*^2^*_adj_*) were obtained by nonlinear least-squares regression. Models were fitted separately to each replicate (*n* = 3), and the resulting parameter estimates were reported as mean ± standard deviation (SD). All parameter values and SD ≤ 10^−2^ were formatted in scientific notation to avoid precision loss from decimal rounding. Model performance was evaluated using *R*^2^*_adj_*, the model selection criterion (MSC), and Akaike’s information criterion (AIC). Higher *R*^2^*_adj_* and MSC values and lower AIC values were considered indicative of better model performance; an MSC value greater than 2 was considered a general guideline for a good fit [19].

#### 2.5.2. Molecular Ion Detection

Molecular ion detection was performed by Gas Chromatography–Mass Spectrometry (GC-MS) using a Thermo Scientific TRACE 1310 Gas Chromatograph coupled to a Thermo Scientific ISQ LT single-quadrupole Mass Spectrometer (Thermo Fisher Scientific, Austin, TX, USA), as previously reported [12,16]. HBA release samples collected after 48 h of release at pH 7.4 were acidified to pH 1.2, extracted with ethyl ether, recrystallized, and dried for 24 h. The dried extracts were redissolved in methanol, filtered through 0.45 μm PTFE syringe filters, and transferred to short-thread ND9 vials for GC–MS analysis.

Chromatographic separation was performed using a Thermo Scientific TG-SQC column. Helium was used as the carrier gas at a flow rate of 1.5 mL/min. The injector temperature was set to 250 °C. The oven temperature was initially maintained at 40 °C for 1 min, followed by a temperature ramp of 40 °C/min to 280 °C, where it was held for 10 min. The transfer line and ion source temperatures were maintained at 240 °C and 210 °C, respectively. Samples were injected in splitless mode with an injection volume of 3 μL. Mass spectra were acquired in electron ionization (EI) mode over a mass-to-charge ratio range of 40–550 *m*/*z*.

## 3. Results

### 3.1. Synthesis and Polymerization of MBA Monomers

MBA monomers were individually synthesized via esterification, as previously reported (Figure S1) [11]. The highest product yields obtained were 65 ± 10%, 86 ± 26% and 48 ± 7% for *o*-MBA, *m*-MBA and *p*-MBA respectively (Table 1).

Free-radical polymerization of the MBA monomers was subsequently performed using AIBN as the initiator, following the previously reported method [11]. The resulting polymer yields were 92 ± 13%, 76 ± 9.0% and 83 ± 7.0% for poly(*o*-MBA), poly(*m*-MBA), and poly(*p*-MBA), respectively (Table 2), confirming the successful conversion of monomers into polymers.

### 3.2. Structural and Spectroscopic Characterization of HBA Isomers, MBA Monomers, and Polymers

#### 3.2.1. ^1^H-NMR and FTIR-ATR Structural Confirmation

The synthesis and structural identity of the MBA monomers were initially confirmed by ^1^H NMR spectroscopy (spectra available in the Supplementary Materials, Figure S2a–c). The resulting spectra showed the characteristic signals of the vinyl protons of the methacrylate group, consistent with previously published data [11,20,21], along with the distinctive aromatic splitting patterns corresponding to the disubstituted rings in the *ortho*, *meta*, and *para* positions. Taken together, these spectral characteristics unequivocally confirm the successful formation of the monomer.

**Figure 1:**
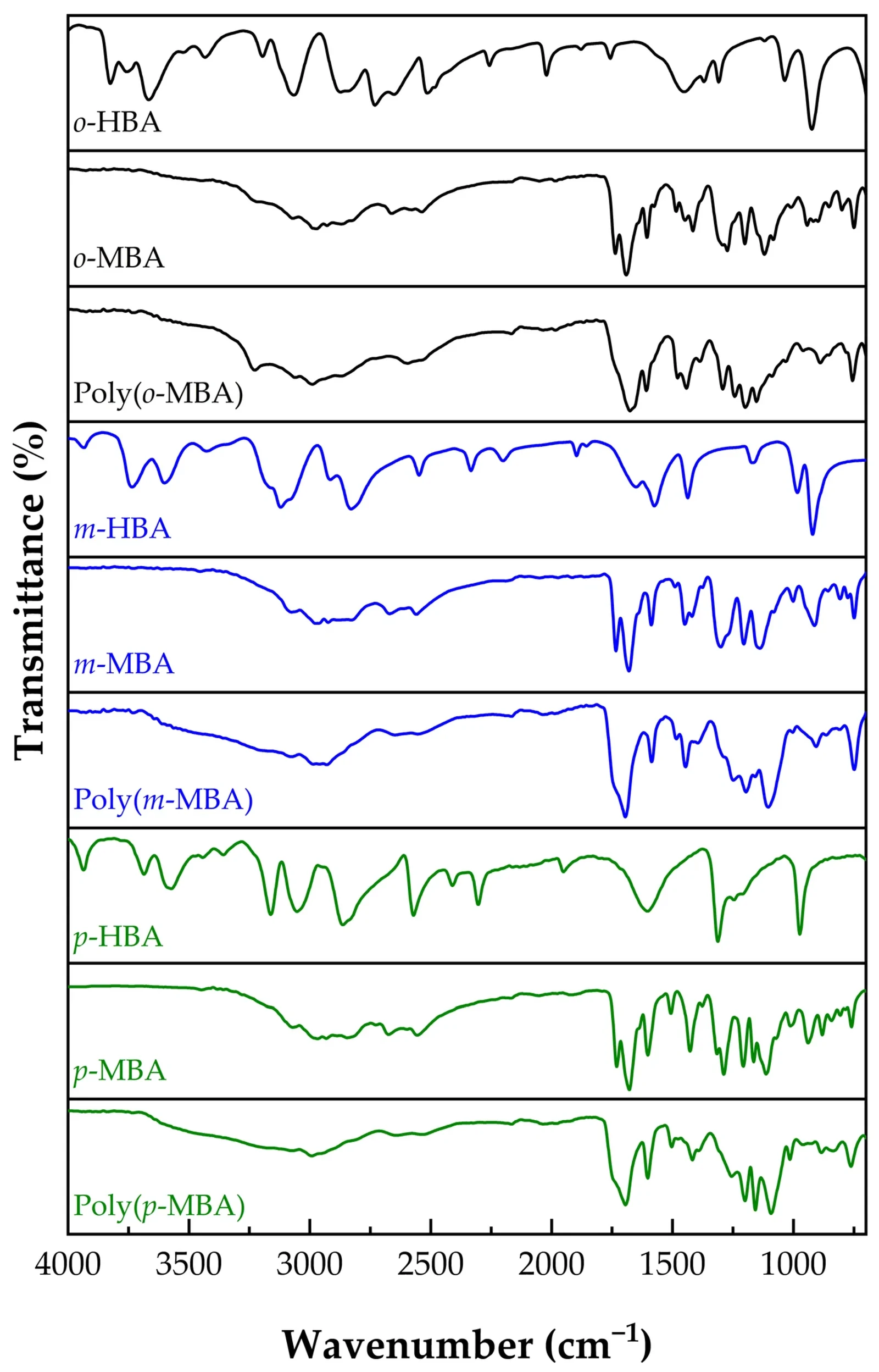
FTIR-ATR spectra of *o*-HBA, *m*-HBA, *p*-HBA, and their corresponding monomers and polymers. Colors indicate substitution patterns: *ortho* (black), *meta* (blue), and *para* (green). MBA: methacryloyloxybenzoic acid; HBA: hydroxybenzoic acids.

To evaluate the successful synthesis of the MBA monomers, their FTIR-ATR spectra were compared against those of the starting precursors (HBA isomers) (Figure 1). This comparative analysis provided clear traceability of the chemical transformation. Specifically, the conversion was evidenced by the emergence of new characteristic signals in the MBA spectra at ~3072, ~2976, ~1680, ~1642, and ~1273 cm^−1^, corresponding to O–H stretch, -CH_3_ (C-H) stretch, strong C=O stretch (acid), H_2_C=C, and C-O, respectively. The appearance of these methacrylate-associated bands confirms the successful esterification of the HBA precursors. Furthermore, to definitively establish the molecular structure, aromatic substitution patterns, and overall purity of the products, ^1^H NMR spectroscopy was performed, which conclusively corroborated the synthesis of the three monomers (Figure S2).

For the ester signal, the *m*- and *p*-MBA monomers showed bands at 1734 and 1730 cm^−1^, respectively. In contrast, the *o*-MBA ester signal appears shifted to 1776 cm^−1^. Aromatic substitution patterns were confirmed by characteristic bands: ~749 cm^−1^ for *ortho*, ~749/807/913 cm^−1^ for *meta*, and ~760 cm^−1^ for *para*-disubstituted rings, further supporting MBA monomer formation. Overall, the ^1^H-NMR and FTIR-ATR analyses confirm the differential positional isomerism of the MBA monomers.

#### 3.2.2. Spectral FTIR-ATR Signatures of MBA Polymers

The MBA polymers’ FTIR-ATR spectral data are summarized in Table 2 and Figure 1. The conversion of monomers to polymers is evidenced by broadening and overlap of characteristic signals and by the loss of the ~1642 cm^−1^ signal corresponding to the vinyl stretching band (H_2_C=C), which indicates polymer backbone formation, consistent with previous reports [22,23]. Additionally, signals at ~1680 and 700–950 cm^−1^ indicate the carbonyl and aromatic substitution integrity in the polymer.

#### 3.2.3. UV-Vis Absorption Profiles and Band Shifts

**Figure 2:**
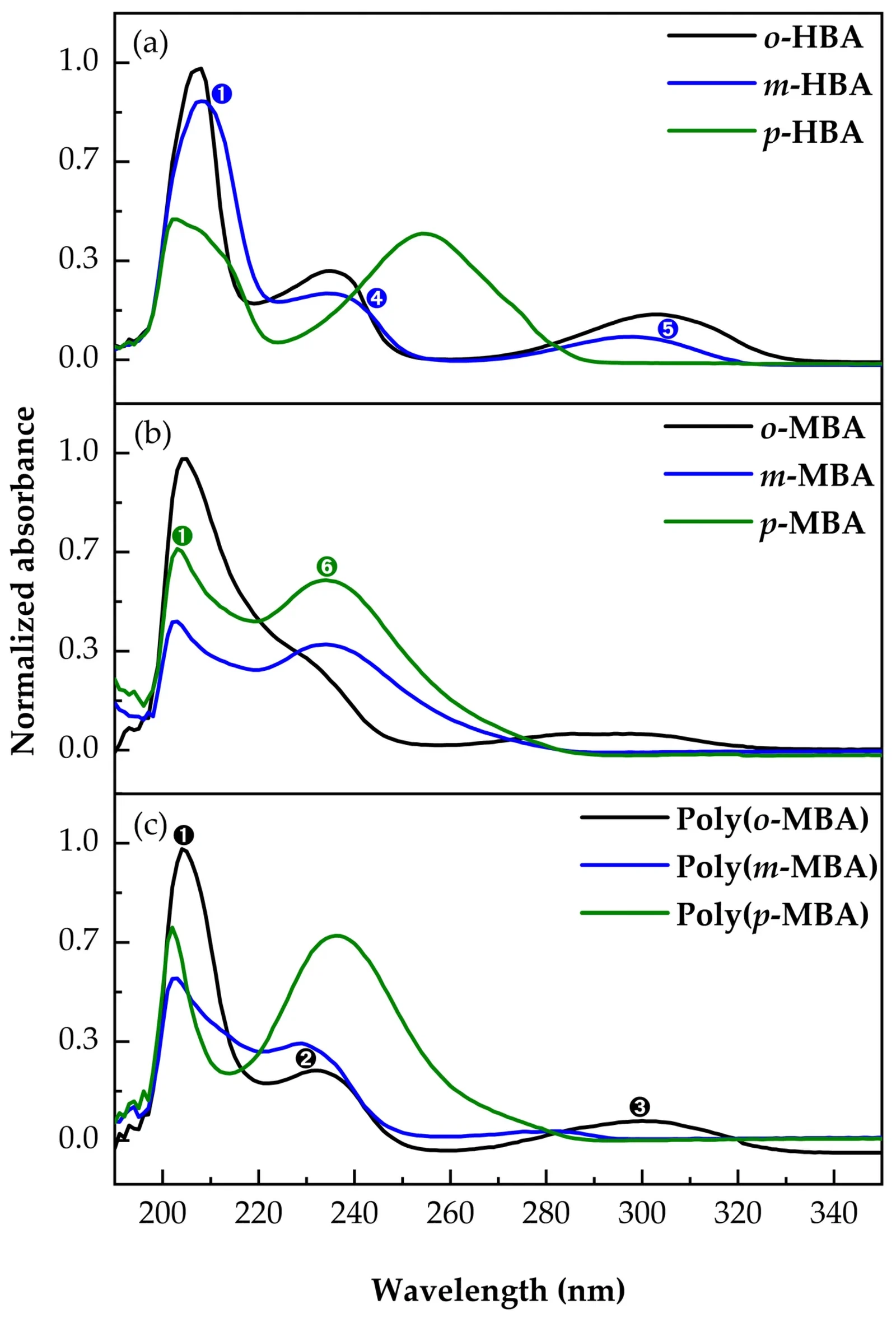
UV spectra of (**a**) HBA isomers, (**b**) MBA monomers, and (**c**) MBA polymers. Characteristic absorption bands and absorption maxima (p_1_–p_6_) are indicated. Colors denote substitution patterns: *ortho* (black), *meta* (blue), and *para* (green). Filled circles mark the principal absorption maxima assigned for each compound. MBA: methacryloyloxybenzoic acid; HBA: hydroxybenzoic acids.

To characterize the UV absorption behavior of HBA isomers, MBA monomers, and MBA polymers, spectra were recorded over the range of 190–340 nm (Figure 2). To account for solvent absorption near its UV cutoff (205 nm), rigorous baseline correction was applied to pure ethanol as a blank in matched quartz cuvettes. In Figure 2, a maximum UV absorption band (p1) was identified for each series: the *ortho* derivatives absorbed at 208, 205, and 204 nm; the *meta* derivatives at 208, 203, and 203 nm; and the *para* derivatives at 203, 203, and 202 nm (for HBA, MBA monomer, and polymer, respectively). These results indicate minimal shifts in the absorption maxima following methacrylation and polymerization.

Beyond the main band, *o*-HBA shows two secondary transitions at 235 nm (p2) and 303 nm (p3), attributed to the aromatic chromophore’s π→π* and n→π* transitions, respectively [24,25]. After methacrylation, both bands change notably in *o*-MBA: p2 attenuates into a broad shoulder, and the relative intensity of p3 decreases significantly, indicating that introducing the methacrylate group alters the local electronic environment of the phenolic moiety. These effects are consistent with previously described spectroscopic and computational studies [26,27], which demonstrate that the unique intramolecular hydrogen bond in *ortho*-HBA derivatives largely determines their electronic delocalization and conformational rigidity; consequently, the disruption of this intramolecular bond during esterification profoundly modifies the local microenvironment and the resulting resonance. Similarly, polymerization partially modifies the spectral profile of poly(*o*-MBA), where peaks p2 and p3 reappear as distinct bands at 232 and 300 nm, respectively, changes that likely reflect alterations in the local microenvironment along the polymer backbone.

Regarding the *meta* isomer, *m*-HBA exhibits secondary bands at 235 nm (p4) and 298 nm (p5). Following methacrylation and subsequent polymerization, band p5 is no longer clearly resolved, whereas p4 persists and shifts slightly to 234 nm in *m*-MBA and to 228 nm in poly(*m*-MBA). The attenuation of band p5 highlights this transition’s sensitivity to substitution of the hydroxyl group at the *meta* position.

Finally, in the *para* series, *p*-HBA shows a secondary band at 254 nm (p6) that shifts hypsochromically after methacrylation, appearing at 234 nm in *p*-MBA and 236 nm in poly(*p*-MBA), accompanied by changes in band intensity. This blue shift is consistent with a modification in the electronic character of the oxygenated substituent following esterification, which affects its resonance interaction with the aromatic ring. Overall, the spectra confirm that the central aromatic chromophore is preserved during methacrylation and polymerization, as evidenced by the persistence of the main absorption bands across all derivatives.

#### 3.2.4. Apparent Polymer Molecular Weight Profiles by Static Light Scattering (SLS)

To estimate the apparent molecular weight (*M_W_*) of the MBA polymers, Static Light Scattering (SLS) measurements were performed and analyzed using Debye plots. The apparent *M_W_* of the synthesized polymers differed substantially among isomers: poly(*o*-MBA) exhibited the lowest apparent *M_W_* at 86,750 ± 19,367 Da, while poly(*p*-MBA) and poly(*m*-MBA) showed considerably higher values of 201,667 ± 4,472 Da and 258,000 ± 35,300 Da, respectively. The reported molecular weights correspond to apparent values derived from static intensity measurements.

These marked differences in macromolecular weight likely stem from steric hindrance during free-radical polymerization, where proximity of the *ortho*-substituent to the methacrylate group may favor early chain termination [28]. Consequently, the distinct release kinetics observed across the series are best understood as reflecting the coupled physical and chemical effects of the synthesized polymers [29]. Because lower molecular weight intrinsically influences matrix density, chain entanglement, and hydration behavior, the release profiles represent the combined contribution of both positional isomerism and macromolecular dimensions rather than structural isomerism alone [28,29].

### 3.3. Biological Efficacy and Biocompatibility

#### 3.3.1. Fibroblast Cell Viability and Cytotoxicity Evaluation

**Figure 3:**
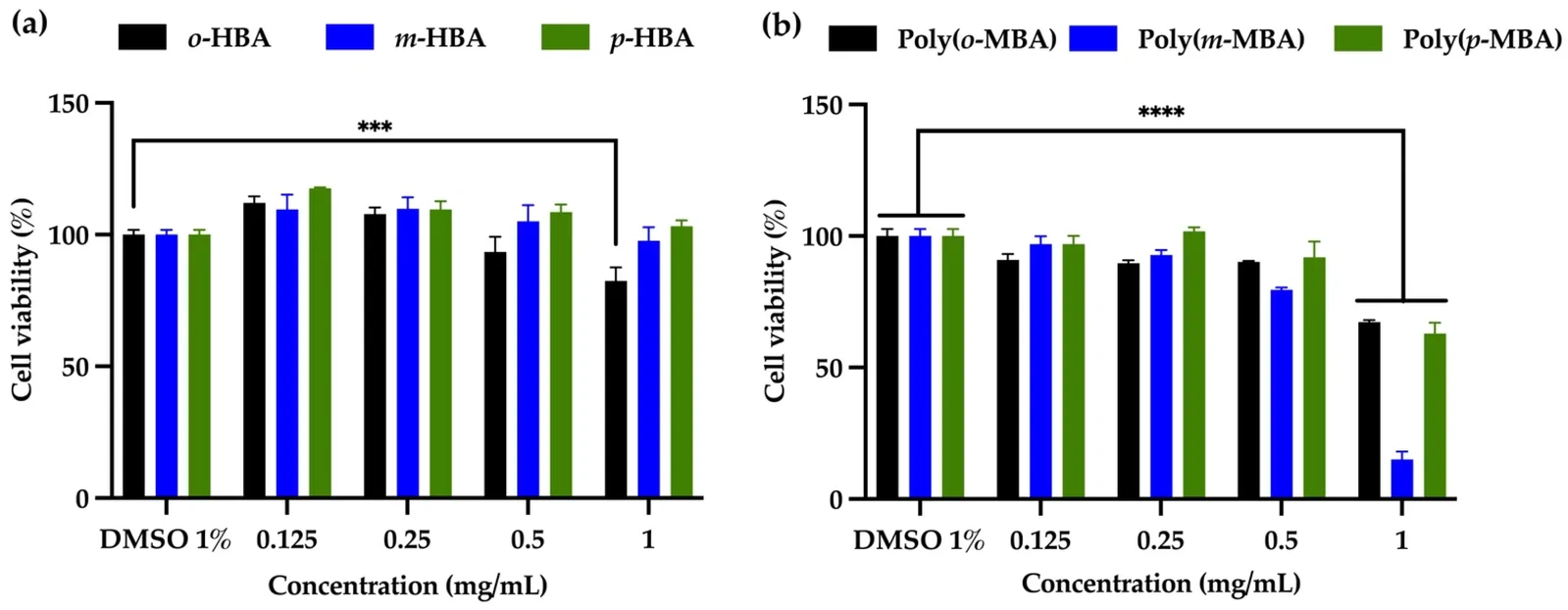
Effect of (**a**) HBA isomers and (**b**) MBA polymers on the viability of BALB/3T3 mouse fibroblasts at concentrations ranging from 0.125 to 1 mg/mL. A 1% (*v*/*v*) DMSO solution was used as the vehicle control and defined as 100% viability. Data are expressed as mean ± standard deviation (*n* = 3). Statistical significance was determined by two-way ANOVA followed by Tukey’s multiple-comparisons test. (*** *p* < 0.001, **** *p* < 0.0001 compared to the control). HBA: hydroxybenzoic acids; MBA: methacryloyloxybenzoic acid.

Because the reduced ionization of MBA polymers and HBA isomers in aqueous media at low to neutral pH limits their solubility [11], DMSO was incorporated to facilitate compound dissolution during biological characterization. Cell viability was evaluated under controlled conditions using an MTT assay in direct-contact cultures of BALB/3T3 mouse fibroblasts at concentrations of 1, 0.5, 0.25, and 0.125 mg/mL, corresponding to DMSO concentrations of 1%, 0.5%, 0.25%, and 0.125% (*v*/*v*), respectively, for both MBA polymers and HBA isomers [13]. After 24 h of exposure, none of the HBA isomers reduced cell viability below 70% across the tested concentration range (Figure 3a), consistent with previous reports [10]. In contrast, MBA polymers induced a concentration-dependent reduction in cell viability (Figure 3b), with poly(*m*-MBA) exhibiting the strongest cytotoxic effect at 1 mg/mL.

MBA polymers exhibited statistically significant differences relative to the DMSO control; however, only the highest concentration tested (1 mg/mL) exceeded the cytotoxicity threshold established by ISO 10993-5:2009 [30]. Cell viability was 67.3% for poly(*o*-MBA), 15.1% for poly(*m*-MBA), and 62.9% for poly(*p*-MBA). Accordingly, poly(*m*-MBA) exhibited the greatest cytotoxic effect, whereas poly(*o*-MBA) and poly(*p*-MBA) also showed cytotoxic effects, with cell viability below 70%.

#### 3.3.2. Planktonic Antibacterial Activity Assay

**Figure 4:**
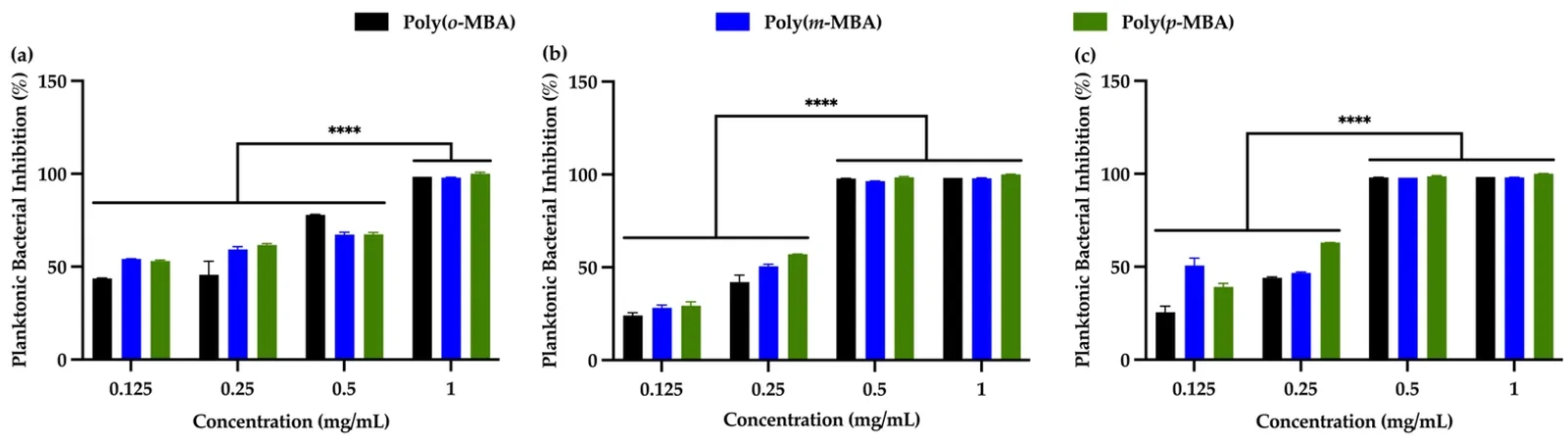
Bacterial inhibition assay by poly(*o*-MBA), poly(*m*-MBA), and poly(*p*-MBA) at concentrations ranging from 0.125 to 1 mg/mL against (**a**) *S. aureus*, (**b**) *E. coli*, and (**c**) *P. aeruginosa*. A 1% (*v*/*v*) DMSO solution was used as the vehicle control and defined as 0% inhibition. Data are expressed as mean ± SD (*n* = 3). Statistical significance was determined by two-way ANOVA followed by Tukey’s multiple-comparisons test. (**** *p* < 0.0001 compared to the control). MBA: methacryloyloxybenzoic acid.

HBA isomers have demonstrated antimicrobial activity [31,32,33]. To evaluate the antimicrobial potential of MBA polymers, planktonic bacterial inhibition assays were performed using polymer concentrations ranging from 0.125 to 1 mg/mL against Gram-positive (*S. aureus*) and Gram-negative (*E. coli* and *P. aeruginosa*) bacteria. Because of the limited aqueous solubility of the polymers, 1% DMSO was used to facilitate dissolution. Complete growth inhibition was achieved at 1 mg/mL for all MBA polymers against all tested strains (Figure 4a–c). Notably, *E. coli* and *P. aeruginosa* were also fully inhibited at 0.5 mg/mL (Figure 4b,c), whereas *S. aureus* exhibited only partial inhibition at the same concentration, suggesting lower susceptibility to the polymers (Figure 4a).

### 3.4. In Vitro HBA Release Profiles and Kinetic ModelingHBA Isomers Release Profiles

The release of HBA isomers from compressed MBA polymer solid dosage forms was quantified using a standardized compendial method established in the *Pharmacopoeia of the United Mexican States* [17]. The identity of the released HBA isomers was further confirmed by molecular ion detection (Figure S7). Release profiles were evaluated over 48 h at 37 °C in PBS under physiologically relevant pH conditions.

The matrix-matched calibration curves showed high coefficients of determination across the three simulated dissolution media, with (*R*^2^) values ranging from 0.9988 to 0.9994. The *%RSD* values for the response factor ranged from 1.3138% to 1.9406% (%RSD < 2), indicating low relative variability in the analytical response. The slopes, intercepts, response factors, and their associated variability are summarized in Table S2, while the individual calibration curves are provided in Figures S4–S6. Together, these results support the linearity and consistency of the HPLC response over the evaluated concentration range in the three dissolution media.

**Figure 5:**
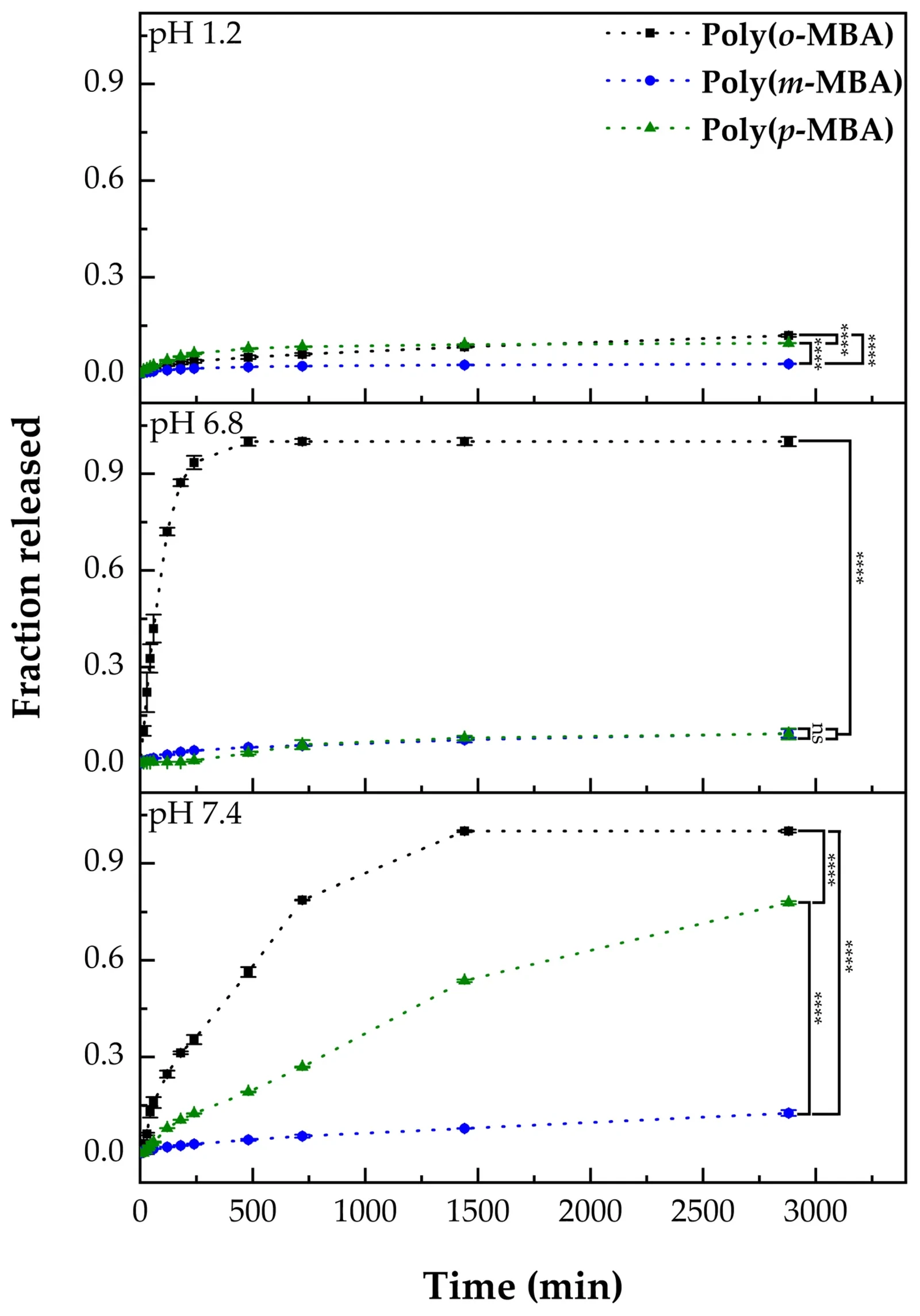
Cumulative fraction of HBA released from MBA polymer-based solid dosage forms as a function of time (0–48 h) at simulated physiological pH values of 1.2 (gastric fluid), 6.8 (intestinal fluid), and 7.4 (plasma). Release studies were conducted in aqueous buffer without organic solvents. Data are expressed as mean ± SD (*n* = 3). Statistical significance was determined by two-way ANOVA followed by Tukey’s multiple-comparisons test. (**** *p* < 0.0001). MBA: methacryloyloxybenzoic acid; HBA: hydroxybenzoic acid.

Poly(*o*-MBA) demonstrated the most pronounced release profile among the three polymers. At gastric pH (1.2), release remained below 11% throughout the 48-h period, consistent with limited release under acidic conditions (Figure 5). As pH increased to intestinal conditions (6.8), *o*-HBA release rose steeply from the onset, reaching a plateau of 100% within only 8 h. At physiological pH (7.4), the same 100% plateau was ultimately achieved but required 24 h (three times longer than at pH 6.8), indicating that while complete release is attainable at both near-neutral pH values, the faster release at pH 6.8 is consistent with accelerated ester hydrolysis and/or matrix erosion under intestinal conditions, although polymer mass loss and ester cleavage in the remaining polymer were not directly measured. At 48 h, the cumulative release from poly(*o*-MBA) differed significantly from that of the other two polymers at all three pH conditions (**** *p* < 0.0001). Within this polymer, the 48-h release differed significantly between pH 1.2 and each near-neutral condition (pH 6.8 and 7.4; **** *p* < 0.0001), but not between pH 6.8 and 7.4. These results highlight the distinct release profile of poly(*o*-MBA) under acidic versus near-neutral conditions.

Poly(*m*-MBA) exhibited a distinct pH threshold-dependent behavior with a markedly different release profile at each condition. At gastric pH (1.2), cumulative release remained at ~3%, with a faster initial rate during the first 8 h followed by a plateau, suggesting an initial release of surface-accessible material, followed by possible diffusion limitation (Figure 5 and Figure S8). At intestinal pH (6.8 and 7.4), release also remained below 13%; however, a distinct 5-h lag phase (Figure S8) preceded any measurable release, after which release increased slowly. At the 48-h endpoint, release from poly(*m*-MBA) differed significantly from that of the other polymers at all pH conditions (**** *p* < 0.0001), except for the comparison with poly(*p*-MBA) at pH 6.8. For poly(*m*-MBA), significant differences were observed between all pairs of pH conditions (**** *p* < 0.0001).

At gastric pH (1.2) and intestinal pH (6.8), *p*-HBA release remained below 10% throughout the 48-h period. In contrast, at physiological pH (7.4), poly(*p*-MBA) displayed a gradual, sustained release profile, achieving ~77% cumulative release by 48 h without reaching a plateau, suggesting that poly(*p*-MBA) release would likely continue beyond the observation window and indicating that pH 7.4 is the condition that most favors *p*-HBA release from this polymer. At 48 h, release from poly(*p*-MBA) differed significantly from that of poly(*o*-MBA) at all three pH conditions and from that of poly(*m*-MBA) at pH 1.2 and 7.4 (**** *p* < 0.0001). The difference between poly(*p*-MBA) and poly(*m*-MBA) at pH 6.8 was not statistically significant. Comparisons across pH conditions showed significant differences between pH 7.4 and both pH 1.2 and 6.8 (**** *p* < 0.0001), whereas the difference between pH 1.2 and 6.8 was not significant.

**Figure 6:**
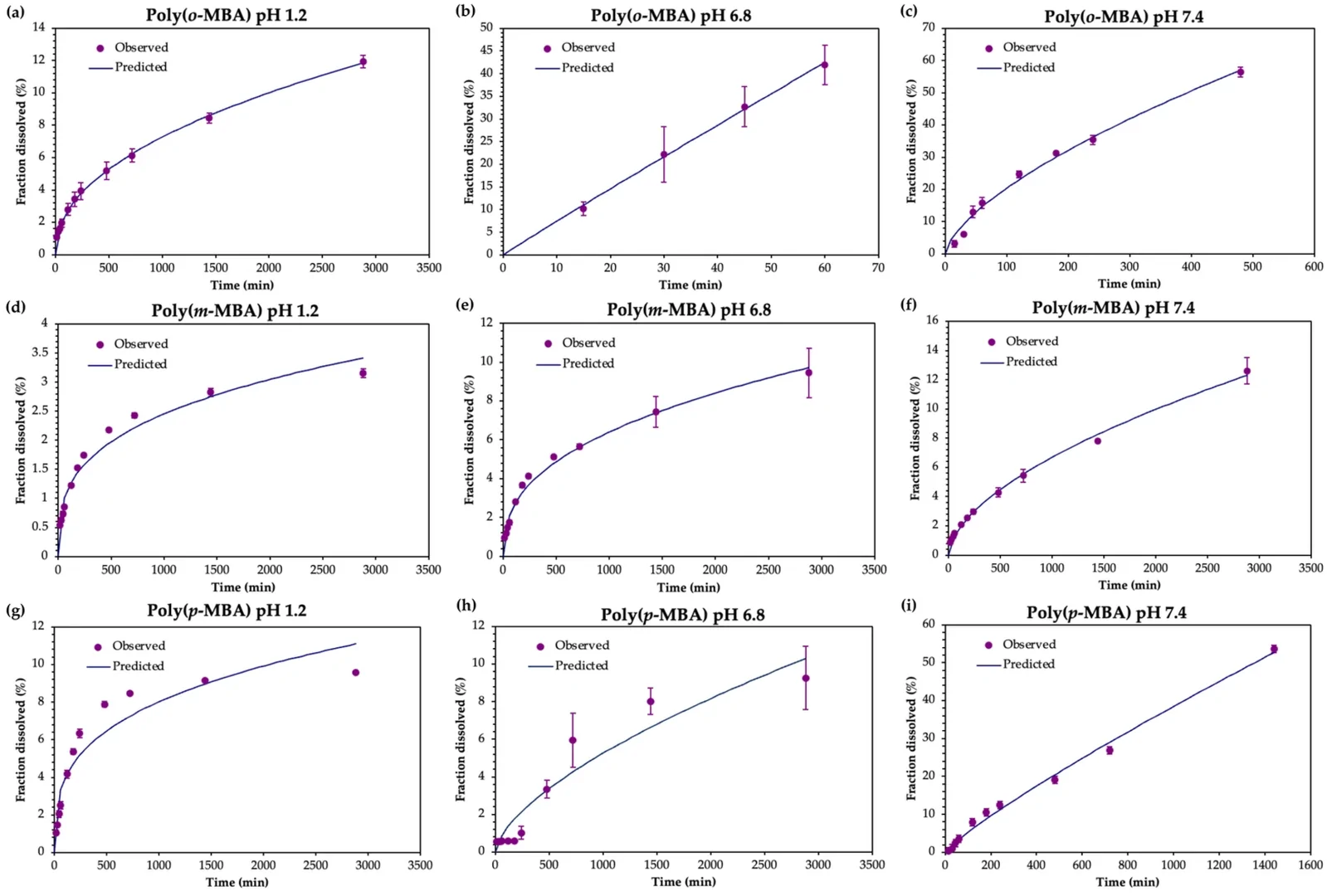
Kinetics of HBA released from MBA polymers at pH 1.2, 6.8, and 7.4. Panels (**a**–**c**), (**d**–**f**), and (**g**–**i**) correspond to poly(*o*-MBA), poly(*m*-MBA), and poly(*p*-MBA), respectively. These findings indicate that the Korsmeyer–Peppas model provided the best overall fit for all MBA polymers across all three pH conditions according to *R^2^_adj_*, MSC and AIC. Symbols represent experimental data, and solid lines represent the fitted curves obtained using the most suitable kinetic model. Data are expressed as mean ± SD (*n* = 3). MBA: methacryloyloxybenzoic acid.

Mathematical modeling of release kinetics is an important tool for characterizing the release profiles of active compounds, estimating relevant parameters, and evaluating the ability of different models to describe experimental release profiles [19,34]. In this study, Zero-order, First-order, Higuchi, and Korsmeyer–Peppas models (Figure 6 and Table S1) were evaluated to characterize the release kinetics of the HBA isomers from the MBA polymers under simulated gastric (pH 1.2), intestinal (pH 6.8), and physiological (pH 7.4) conditions.

Among the models evaluated, the Korsmeyer–Peppas model yielded the highest adjusted coefficients of determination (*R*^2^*_adj_*) for poly(*o*-MBA) at pH 1.2 (0.99), pH 6.8 (0.97), and pH 7.4 (0.98); for poly(*m*-MBA) at pH 1.2 (0.96), pH 6.8 (0.98), and pH 7.4 (0.99); and for poly(*p*-MBA) at pH 1.2 (0.86), pH 6.8 (0.90), and pH 7.4 (0.99) (Table S1). These findings indicate that the Korsmeyer–Peppas model provided the best overall fit across all three pH conditions according to *R*^2^*_adj_*, MSC and AIC (Table S1).

The release exponent (*n*) obtained from the Korsmeyer–Peppas model was used to infer the predominant drug-release mechanism based on the criteria reported by Ritger and Peppas [34]. For cylindrical matrices, *n* ≤ 0.45 suggests a trend consistent with Fickian diffusion, 0.45 < *n* < 0.89 indicates anomalous (non-Fickian) transport involving coupled diffusion and polymer relaxation processes, *n* = 0.89 indicates Case II transport, and *n* > 0.89 indicates super Case II transport. Fickian diffusion was indicated for poly(*m*-MBA) at pH 1.2 (*n* = 0.31) and pH 6.8 (*n* = 0.39), and for poly(*p*-MBA) at pH 1.2 (*n* = 0.30). Anomalous transport was indicated for poly(*o*-MBA) at pH 1.2 (*n* = 0.46) and pH 7.4 (*n* = 0.65), poly(*m*-MBA) at pH 7.4 (*n* = 0.57), and poly(*p*-MBA) at pH 6.8 (*n* = 0.63) and pH 7.4 (*n* = 0.86). The latter value approaches the threshold for Case II transport. A value consistent with Case II transport was obtained for poly(*o*-MBA) at pH 6.8 (*n* = 0.90).

For poly(*m*-MBA) and poly(*p*-MBA), the release exponent increased with pH, from 0.31 to 0.39 and 0.57 for poly(*m*-MBA), and from 0.30 to 0.63 and 0.86 for poly(*p*-MBA), at pH 1.2, 6.8, and 7.4, respectively. This trend suggests a shift from predominantly Fickian release toward transport behavior involving a greater contribution from polymer relaxation or other matrix-related processes [19,35]. However, because the Korsmeyer–Peppas exponent (*n*) is an empirical fitting parameter, it indicates qualitative release trends but does not establish definitive physical transport regimes or determine the specific contributions of swelling and polymer dissolution. In contrast, poly(*o*-MBA) showed no monotonic change in *n* with increasing pH. Notably, its values at pH 1.2 (*n* = 0.46) and pH 6.8 (*n* = 0.90) lie close to or just beyond the conventional boundaries separating Fickian, anomalous, and Case II transport regimes, respectively, and should therefore be interpreted with appropriate caution.

## 4. Discussion

The spectroscopic changes observed after methacrylation and polymerization provide insight into the electronic and conformational differences among the MBA derivatives. The ester signals of the m- and p-MBA monomers are observed at 1734 and 1730 cm^−1^, whereas the o-MBA ester signal is shifted to 1776 cm^−1^. Rather than concluding that a mechanism has been established, we hypothesize that this marked shift reflects an electronic perturbation and steric hindrance associated with the close spatial proximity of the ortho-carboxyl group. This interpretation is consistent with the well-documented spectral behavior of other ortho-substituted phenolic esters, where steric clash forces the ester group out of the aromatic plane, thereby reducing resonance and increasing the carbonyl stretching frequency [36].

The ^1^H NMR spectra provided complementary evidence of differences in the chemical environments of the aromatic and methacrylate protons among the MBA monomers. The aromatic signals exhibited patterns consistent with the different substitution patterns of the aromatic ring [37,38,39]. The carboxylic acid proton signal was observed at δ 12.9 ppm for *m*-MBA and *p*-MBA but was not clearly detected for *o*-MBA. This absence does not necessarily indicate that the carboxylic acid group is absent. The chemical shift of its proton may be influenced by the electronic environment, including the effects of nearby electronegative atoms and the magnetic anisotropy of the carbonyl group. Furthermore, its detectability may be affected by sample concentration, proton exchange, hydrogen bonding, and experimental conditions, which can cause signal broadening or reduce signal intensity [40,41,42]. Therefore, the absence of a clearly resolved signal in the *o*-MBA spectrum cannot be attributed to a single cause based on the available data. Although ^1^H NMR alone does not provide definitive structural confirmation of the positional isomers, the observed differences in chemical shifts and signal patterns support the successful preparation of chemically distinct MBA monomers. Together with the complementary spectroscopic characterization, these results support the formation of the corresponding MBA polymers.

The ^1^H NMR and FTIR-ATR results support the synthesis and structural assignment of the MBA monomers used as precursors for polymerization. These findings are consistent with the proposed design of the MBA-based materials as PPs, in which the HBA-derived moiety is covalently attached to the polymer through a hydrolysable ester linkage [11,12]. The release experiments further investigate the release of the corresponding parent HBA from the PP materials. Nevertheless, direct ^1^H NMR characterization of the MBA polymers was not performed, and the monomer spectra alone cannot establish the complete chemical structure of the polymeric products. This limitation should be considered when interpreting the structural evidence. Additional characterization of the polymers using suitable complementary analytical techniques would help further substantiate their proposed structures in future studies.

The UV–Vis spectra revealed changes in band positions, shapes, and relative intensities following methacrylation and polymerization, while the persistence of the main absorption bands indicated that the aromatic chromophore was retained. The spectral changes differed among the positional isomers, suggesting that the position of the substituents influences the optical response of the resulting derivatives. In particular, the changes observed for the *ortho* isomer may be related to its distinct local electronic environment, which has been associated in previous studies with intramolecular hydrogen bonding in salicylic acid derivatives [26,27]. However, the present spectra do not directly establish changes in hydrogen bonding, electronic delocalization, or conformational organization. Therefore, these features are considered possible explanations for the observed spectral differences rather than experimentally confirmed mechanisms.

The lower cell viability observed with poly(*m*-MBA) at 1 mg/mL was unexpected, given that the corresponding *m*-HBA isomer did not exhibit cytotoxicity at the tested concentration and that all three polymers share the same methacrylate-derived backbone. This finding suggests that factors beyond the *m*-HBA moiety alone, including differences in polymer structure or physicochemical properties, may contribute to the observed response. Among these, poly(*m*-MBA) exhibited the highest apparent *M_W_* of the three polymers (258,000 ± 35,300 Da vs. 201,667 ± 4,472 Da for poly(*p*-MBA) and 86,750 ± 19,367 Da for poly(*o*-MBA)), which may contribute to, but does not fully account for, the observed effect, as poly(*p*-MBA)’s intermediate molecular weight was not associated with a comparable reduction in viability. Residual monomer and residual AIBN initiator were not quantified in this study and represent a limitation to be addressed in future work. All three polymers were purified using an identical protocol, making differential solvent residues a less likely contributor, although this was not directly measured. Additionally, the solubility of the parent HBA isomers and the solubility and dispersion behavior of the corresponding polymers were not determined in this study. Therefore, their potential contributions to the observed cytotoxicity and release remain unclear. Future work should characterize polymer dispersion in the relevant biological media (e.g., by DLS and PDI analysis) and assess whether experimental sink conditions are maintained during release experiments. Overall, these findings indicate that the cell viability of MBA polymers is concentration- and isomer-dependent under the tested conditions, rather than broadly favorable. This assessment was limited to a single fibroblast line (BALB/3T3) at a single 24 h endpoint, and the 70% viability threshold (ISO 10993-5:2009 [30]) was applied as an interpretative benchmark rather than as a full standard-compliant evaluation, which would require standardized extracts, multiple cell lines, and additional time points. Biocompatibility claims should therefore be considered preliminary, and further mechanistic characterization is needed before incorporation of these polymers into PDDS can be recommended.

The antibacterial activity observed under DMSO-assisted dispersion conditions indicates that the MBA polymers exhibited planktonic antibacterial activity under the tested conditions, consistent with previous reports [33]. It should be noted that these results represent endpoint measurements of bacterial growth inhibition after 24 h. While this demonstrates a strong antimicrobial effect at the evaluated time point, future studies incorporating time-course growth curves will be necessary to fully characterize the temporal release dynamics and distinguish between delayed growth and sustained complete inhibition. The release studies further suggest that differences in release may contribute to the isomer-dependent behavior of these materials, although this relationship requires further investigation. Collectively, these findings demonstrate concentration- and isomer-dependent inhibition of planktonic bacterial growth against clinically relevant pathogens.

It is important to emphasize that the antibacterial assays conducted in this study evaluate planktonic growth inhibition. While these results confirm the intrinsic antimicrobial capacity of the MBA polymers, this planktonic antibacterial data should not be directly extrapolated to anti-biofilm activity. In the context of implant-associated infections and medical device applications, surface-mediated biological interactions and bacterial adhesion mechanics play a critical role. Future studies should employ specific methods to assess biofilm formation on functional materials and investigate surface-mediated biological interactions, thereby distinguishing planktonic antibacterial activity from the inhibition of surface colonization [43].

Although poly(*o*-MBA) exhibited the lowest apparent *M_W_*, the distinct release profiles observed among the three MBA polymers reflect the combined influence of macromolecular chain size and positional isomerism. While apparent *M_W_* can influence chain entanglements, matrix free volume, diffusion, and swelling in compressed solid dosage forms, substituent-dependent differences in ester accessibility, local polymer microenvironment, and potential electronic effects also contribute to hydrolytic release behavior [28,29]. Because molecular weight and substitution patterns vary simultaneously across the isomer series, these contributions cannot be experimentally decoupled from the present dataset.

Overall, these results highlight the strong influence of positional isomerism and macromolecular size on the release kinetics of MBA polymers. It must be noted that due to the confined volume of the dialysis donor compartment (5 mL), local saturation may transiently occur inside the membrane, meaning strict sink conditions cannot be guaranteed throughout the experiment, particularly at pH 1.2, where HBA solubility is lowest. Nevertheless, because we evaluated all three polymers under identical experimental constraints, the observed variations reflect genuine kinetic differences inherent to their structures. For instance, poly(*m*-MBA) showed the most restricted release across all tested pH values. At gastric pH (1.2), its release remained near zero throughout the 48 h experiment (cumulative fraction of 0.03). Given that poly(*m*-MBA) exhibited minimal HBA release, its behavior appears to be largely independent of medium saturation limits. Rather, these observations point to a combined influence of the aromatic substitution pattern and macromolecular structure on hydrolytic susceptibility across the evaluated pH range.

These divergent release profiles likely reflect variations in polymer hydrophilicity, chain packing, and steric accessibility of the ester linkage governed by substituent position. At gastric pH, the predominantly protonated state of the carboxyl groups (apparent pKa = 5.8) may limit ionization and hydration [11], consistent with the low cumulative release observed for all three polymers (below 11%). At higher pH, the divergent profiles can be described by hypothetical mechanisms operating alongside macromolecular chain length effects: the rapid and near-complete release of poly(*o*-MBA) may be consistent with intramolecular catalytic assistance by the neighboring *ortho*-carboxyl group, potentially involving a geometrically favorable cyclic transition state, as described in the literature for related chemical systems [44,45,46], together with enhanced hydration upon carboxyl ionization (apparent pKa = 5.8) [11]. However, this mechanism remains hypothetical, as it was not directly investigated in the present study. The slower, sustained release profile of poly(*p*-MBA) may be associated with differences in ester accessibility and matrix-mediated transport [47]; however, the release data alone do not establish a specific hydrolysis pathway. Conversely, the *meta*-substitution pattern of poly(*m*-MBA) lacks the neighboring-group intramolecular assistance and direct resonance activation, while potentially unfavorable steric geometry and lower matrix hydration may contribute to its persistently restricted release [48]. Its higher molecular weight (258 kDa), combined with potentially tighter chain packing, may contribute to reduced water accessibility to the hydrolysable ester bonds, offering a plausible explanation for the restricted release observed experimentally [28,29]. Intramolecular catalysis, intermolecular hydrolysis, and steric effects are possible explanations for the observed behavior; however, these mechanisms remain hypothetical and require further experimental investigation. Although mathematical modeling provides a robust quantitative description of HBA release profiles, the Korsmeyer–Peppas exponent (*n*) alone cannot independently resolve the relative physical contributions of polymer swelling, matrix erosion, and solute diffusion. The observed shifts in *n* with pH provide useful empirical indicators of altered release kinetics, but definitive assignment of physical transport mechanisms remains subject to future experimental evaluations of polymer swelling dynamics and mass loss.

These findings also provide a critical interpretive context for the biological activity observed in the planktonic antibacterial activity and cell viability assays. In those experiments, 1% DMSO was incorporated into the medium to overcome the inherently limited aqueous solubility of the MBA polymers as previously reported [49], thereby facilitating compound dissolution and enabling measurable antibacterial and cytotoxic effects for all three polymers, including poly(*m*-MBA). In contrast, the release studies were performed under physiologically relevant aqueous conditions using compressed solid dosage forms in aqueous media rather than fully dissolved polymer solutions, more closely resembling the behavior expected in practical biomedical applications. Under these conditions, poly(*m*-MBA) released only negligible amounts of *m*-HBA across all tested pH values, suggesting that the biological activity observed in solution-based assays may depend largely on DMSO-assisted polymer dispersion rather than on efficient intrinsic hydrolytic susceptibility. Although differences in apparent *M_W_* may influence chain mobility, diffusion, and swelling behavior in fully solvated polymer systems, the markedly distinct pH-dependent release profiles observed among the MBA polymers under solid-state conditions are more consistent with substituent-dependent hydrolytic susceptibility and local polymer microenvironment effects than with molecular weight differences alone. These observations are consistent with the notion that positional isomerism influences release kinetics through differences in ester accessibility, polymer–water interactions, and intramolecular organization.

Taken together, these results establish positional isomerism as a key structural determinant of pH-responsive release kinetics in MBA polymers and underscore the importance of evaluating polymeric prodrugs under formulation-relevant conditions to accurately predict their therapeutic performance.

## 5. Conclusions

In summary, the MBA monomers and polymers were successfully synthesized and structurally characterized by ^1^H-NMR, FTIR-ATR, and UV-Vis analysis. The distinct spectral profiles confirmed the structural differences among the isomers.

At the highest tested concentration (1 mg/mL), poly(*m*-MBA) exhibited the greatest cytotoxic effect, with a cell viability of 15.1%, compared with 67.3% for poly(*o*-MBA) and 62.9% for poly(*p*-MBA). All three polymers reduced cell viability below the 70% threshold established by ISO 10993-5:2009, indicating cytotoxic potential under the tested conditions. In contrast, the HBA isomers did not produce any adverse effects at any of the tested concentrations.

The three MBA polymers demonstrated planktonic antibacterial activity against *S. aureus*, *E. coli*, and *P. aeruginosa* at 1 mg/mL and against *E. coli* and *P. aeruginosa* at 0.5 mg/mL, highlighting their broad-spectrum planktonic antimicrobial activity, particularly against Gram-negative strains.

Finally, the validated HPLC release assays [17] demonstrated marked pH-dependent differences in the release profiles of the MBA polymers. Under higher-pH conditions, poly(*o*-MBA) exhibited rapid and extensive release, poly(*p*-MBA) showed an intermediate release profile, and poly(*m*-MBA) exhibited a limited release. These differences reflect the combined contributions of positional isomerism, apparent molecular weight, and medium pH to the overall release behavior.

Overall, the combination of pH-responsive release and broad-spectrum planktonic antimicrobial activity highlights the potential of MBA polymers as versatile platforms for pharmaceutical and biomedical applications, including localized drug delivery systems and medical-device surfaces. Previous studies have reported water-insoluble polymeric complexes for the controlled release of propranolol and labetalol [14], as well as poly(2MBA)-based grafted systems for sustained salicylic acid release from polymeric films [12].

## Figures and Tables

**Table 1:** Spectral and yield data of MBA monomers.

Monomer	Bond	FTIR-ATR (cm^−1^)	^1^H-NMR (ppm)	Yield (%)
*o*-MBA	*O* − *H*	3072, b	not detected	65 ± 10
	=*C* − *H* (*arom*.)	749, s	8.2–7.1, m (4H)	
	*α* − *CH*_3_ (*C* − *H*)	2972	2.08, t (3H)	
	*C* = 0 (*ester*)	1776		
	*C* = 0 (*acid*)	1691		
	*H*_2_*C* = *C*	1645	6.36, t (1H); 5.78, t (1H)	
	*C* − 0	1273		
*m*-MBA	*O* − *H*	3084, b	12.9, br s (1H)	86 ± 26
	=*C* − *H* (*arom*.)	913, m; 807, s; 749, s	7.4–6.9, m (4H)	
	*α* − *CH*_3_ (*C* − *H*)	2976	2.01, t (3H)	
	*C* = 0 (*ester*)	1734		
	*C* = 0 (*acid*)	1680		
	*H*_2_*C* = *C*	1639	6.3, t (1H); 5.93, t (1H);	
	*C* − 0	1300		
*p*-MBA	*O* − *H*	3072, b	12.9, br s (1H)	48 ± 7
	=*C* − *H* (*arom*.)	760, s	8.1–7.29, m (4H)	
	*α* − *CH*_3_ (*C* − *H*)	2968	2.01, t (3H)	
	*C* = 0 (*ester*)	1730		
	*C* = 0 (*acid*)	1679		
	*H*_2_*C* = *C*	1642	6.31, t (1H); 5.90, t (1H)	
	*C* − 0	1288		

Reported yields represent the average of three independent synthesis batches (*n* = 3). Abbreviations: MBA, methacryloyloxybenzoic acid; FTIR-ATR, Fourier-transform infrared spectroscopy with attenuated total reflectance; arom., aromatic ring; b, broad; m, medium; s, strong; ^1^H NMR, proton nuclear magnetic resonance; br s, broad singlet; s, singlet; d, doublet; dd, doublet of doublets; dt, doublet of triplets; td, triplet of doublets; t, triplet; m, multiplet.

**Table 2:** Characteristic FTIR-ATR bands and yields of MBA polymers.

Polymer	Bond	FTIR-ATR (cm^−1^)	Yield (%)
Poly(*o*-MBA)	*O* − *H*	3227, b	92 ± 13
	=*C* − *H* (*arom*.)	755, s	
	*α* − *CH*_3_ (*C* − *H*)	2989	
	*C* = 0 (*ester*)	Overlap	
	*C* = 0 (*acid*)	1674	
	*C* − 0	1292	
Poly(*m*-MBA)	*O* − *H*	3078, b	76 ± 9.0
	=*C* − *H* (*arom*.)	906, m; 809, s; 748, s	
	*α* − *CH*_3_ (*C* − *H*)	2998	
	*C* = 0 (*ester*)	Overlap	
	*C* = 0 (*acid*)	1695	
	*C* − 0	1253	
Poly(*p*-MBA)	*O* − *H*	3075, b	83 ± 7.0
	=*C* − *H* (*arom*.)	762, s	
	*α* − *CH*_3_ (*C* − *H*)	2991	
	*C* = 0 (*ester*)	Overlap	
	*C* = 0 (*acid*)	1694	
	*C* − 0	1255	

Reported yields represent the average of three independent synthesis batches (*n* = 3). Abbreviations: MBA, methacryloyloxybenzoic acid; FTIR-ATR, Fourier-transform infrared spectroscopy with attenuated total reflectance; arom., aromatic ring; b, broad; m, medium; s, strong.

## Data Availability

The original contributions presented in this study are included in the article/Supplementary Materials.

## Supplementary Materials

The following supporting information can be downloaded at: https://www.mdpi.com/article/10.3390/polym18212789/s1, Figure S1. Synthetic route for the preparation of (b) *o*-MBA, (d) *m*-MBA and (f) *p*-MBA (methacryloyloxy-*o-*, *m-* or *p*-benzoic acid) from the corresponding hydroxybenzoic acid isomers: (a) *o*-, (c) *m*-, and (e) *p*-hydroxybenzoic acid. Colors indicate substitution patterns as follows: *ortho* (black), *meta* (blue), and *para* (green). Red structures correspond to methacrylic anhydride-derived moieties. Figure S2. ^1^H-NMR (400 MHz) spectra of (a) *o*-MBA, (b) *m*-MBA and (c) *p*-MBA dissolved in CDCl_3_. The signals were assigned as follows: δ 12.9 (br s, 1H, carboxylic acid proton; observed for *m*-MBA and *p*-MBA); δ 8.2–7.0 (m, 4H, aromatic protons); δ 6.4–5.7 (two signals, 1H each, alkenyl protons); and δ 2.0 (t, 3H, methyl protons). MBA, methacryloyloxybenzoic acid; br s, broad singlet; s, singlet; d, doublet; dd, doublet of doublets; dt, doublet of triplets; td, triplet of doublets; t, triplet; m, multiplet. Figure S3. Representative HPLC chromatograms of standard solutions (200 µg/mL) of *o*-HBA, *m*-HBA, and *p*-HBA under simulated gastric (pH 1.2), intestinal (pH 6.8), and physiological (pH 7.4) conditions. Panels (a–c), (d–f), and (g–i) correspond to *o*-HBA, *m*-HBA, and *p*-HBA, respectively, while panels (a,d,g), (b,e,h), and (c,f,i) represent pH 1.2, 6.8, and 7.4 conditions, respectively. HBA: hydroxybenzoic acids. Figure S4. Calibration curves of *o*-HBA under simulated gastric (a, pH 1.2), intestinal (b, pH 6.8), and physiological (c, pH 7.4) conditions. Standard solutions were prepared under the same conditions as the analyzed samples. Data are presented as mean ± SD (*n* = 3), and linear regression analysis was performed for each pH condition. HBA: hydroxybenzoic acids. Figure S5. Calibration curves of *m*-HBA under simulated gastric (a, pH 1.2), intestinal (b, pH 6.8), and physiological (c, pH 7.4) conditions. Standard solutions were prepared under the same conditions as the analyzed samples. Data are presented as mean ± SD (*n* = 3), and linear regression analysis was performed for each pH condition. HBA: hydroxybenzoic acids. Figure S6. Calibration curves of *p*-HBA under simulated gastric (a, pH 1.2), intestinal (b, pH 6.8), and physiological (c, pH 7.4) conditions. Standard solutions were prepared under the same conditions as the analyzed samples. Data are presented as mean ± SD (*n* = 3), and linear regression analysis was performed for each pH condition. HBA: hydroxybenzoic acids. Figure S7. Mass spectra of (a) *o*-HBA, (b) *m*-HBA, and (c) *p*-HBA released from poly(*o*-MBA), poly(*m*-MBA), and poly(*p*-MBA), respectively, after 48 h of incubation at pH 7.4. HBA: hydroxybenzoic acids; MBA: methacryloyloxybenzoic acid. Figure S8. Cumulative fraction of HBA released from MBA polymer-based solid dosage forms as a function of time (0–24 h) at simulated physiological pH values of 1.2 (gastric fluid), 6.8 (intestinal fluid), and 7.4 (plasma). Release studies were conducted in aqueous buffer without organic solvents. Data are expressed as mean ± SD (*n* = 3). The y-axis was limited to a maximum released fraction of 0.25 to facilitate visualization of the early-stage release behavior. MBA: methacryloyloxybenzoic acid; HBA: hydroxybenzoic acid. Table S1. Estimated kinetic parameters and goodness-of-fit and model selection criteria for Zero-order, First-order, Higuchi, and Korsmeyer–Peppas models applied to MBA polymer release profiles under different pH conditions. Table S2. Analytical validation parameters for the HPLC quantitative determination of HBA isomers (o-HBA, m-HBA, and p-HBA) across simulated dissolution media according to equations established in the Pharmacopoeia of the United Mexican States.
