# Supplementary material for: Positional Isomerism Influences pH-Responsive Release Kinetics in Methacryloyloxybenzoic Acid Polymeric Prodrugs
Source: Polymers (Basel). 2026 Nov 8;18(21):2789. doi: 10.3390/polym18212789 (PMC13765594; doi:10.3390/polym18212789)
Supplement: Supplementary file 1 [file polymers-18-02789-s001.zip › polymers-4598937-supplementary.pdf]

Positional Isomerism Influences pH-Responsive Release Kinetics in Methacryloyloxybenzoic Acid Polymeric Prodrugs

Hebrón Vázquez-Estudillo [1], Giovanni Palomino-Vizcaino [2], Brisa G. Hernández-Ramírez [1], Lilia A. Hurtado-Ayala [1], Jonathan Vincent Lopez-Baena [1], Teresa G. Rodriguez-Tellez [1], Iván Cordova-Guerrero [1], Lucia E. Azuara-Alvarez [2], Arturo Estolano-Cobián [1], Kenia Palomino-Vizcaino [1], José M. Cornejo-Bravo [1] and Héctor Magaña [1,*]

[1] Faculty of Chemical Sciences and Engineering, Autonomous University of Baja California, University Boulevard No. 14418, Otay Mesa, Tijuana 22390, Mexico; hebron.vazquez@uabc.edu.mx (H.V.-E.); brisa.hernandez@uabc.edu.mx (B.G.H.-R.); lilyhurtado@uabc.edu.mx (L.A.H.-A.); vlopez16@uabc.edu.mx (J.V.L.-B.); rodriguezt1@uabc.edu.mx (T.G.R.-T.); icordova@uabc.edu.mx (I.C.-G.); estolano.arturo@uabc.edu.mx (A.E.-C.); kenia.palomino@uabc.edu.mx (K.P.-V.); jmcornejo@uabc.edu.mx (J.M.C.-B.)

[2] Faculty of Health Sciences, Autonomous University of Baja California, 1000 University Boulevard, Valle de Las Palmas, Tijuana 22260, Mexico; gpalomino@uabc.edu.mx (G.P.-V.); lucia.azuara@uabc.edu.mx (L.E.A.-A.)

* Correspondence: hector.magana@uabc.edu.mx; Tel.: +52-664-293-4810

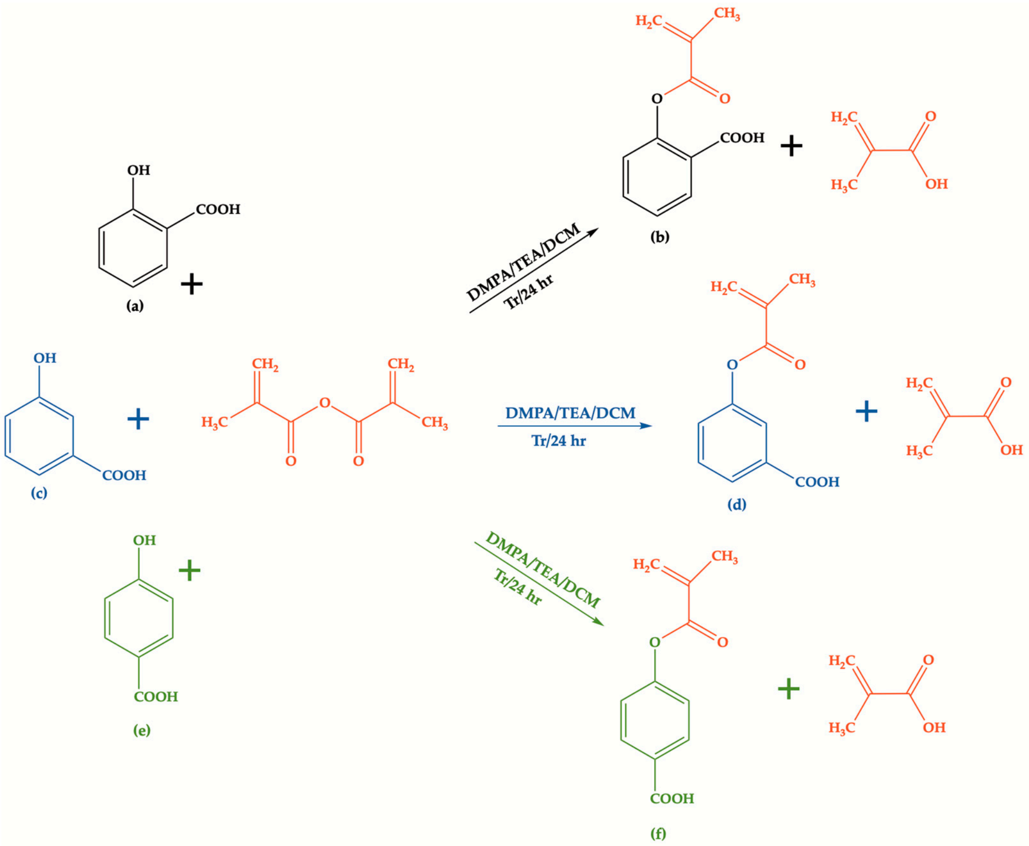

Figure S1. Synthetic route for the preparation of **(b)** *o*-MBA, **(d)** *m*-MBA and **(f)** *p*-MBA (methacryloyloxy-*o*, *m* or *p*-benzoic acid) from the corresponding hydroxybenzoic acid isomers: **(a)** *o*-, **(c)** *m*-, and **(e)** *p*-hydroxybenzoic acid. Colors indicate substitution patterns as follows: *ortho* (black), *meta* (blue), and *para* (green). Red structures correspond to methacrylic anhydride-derived moieties.

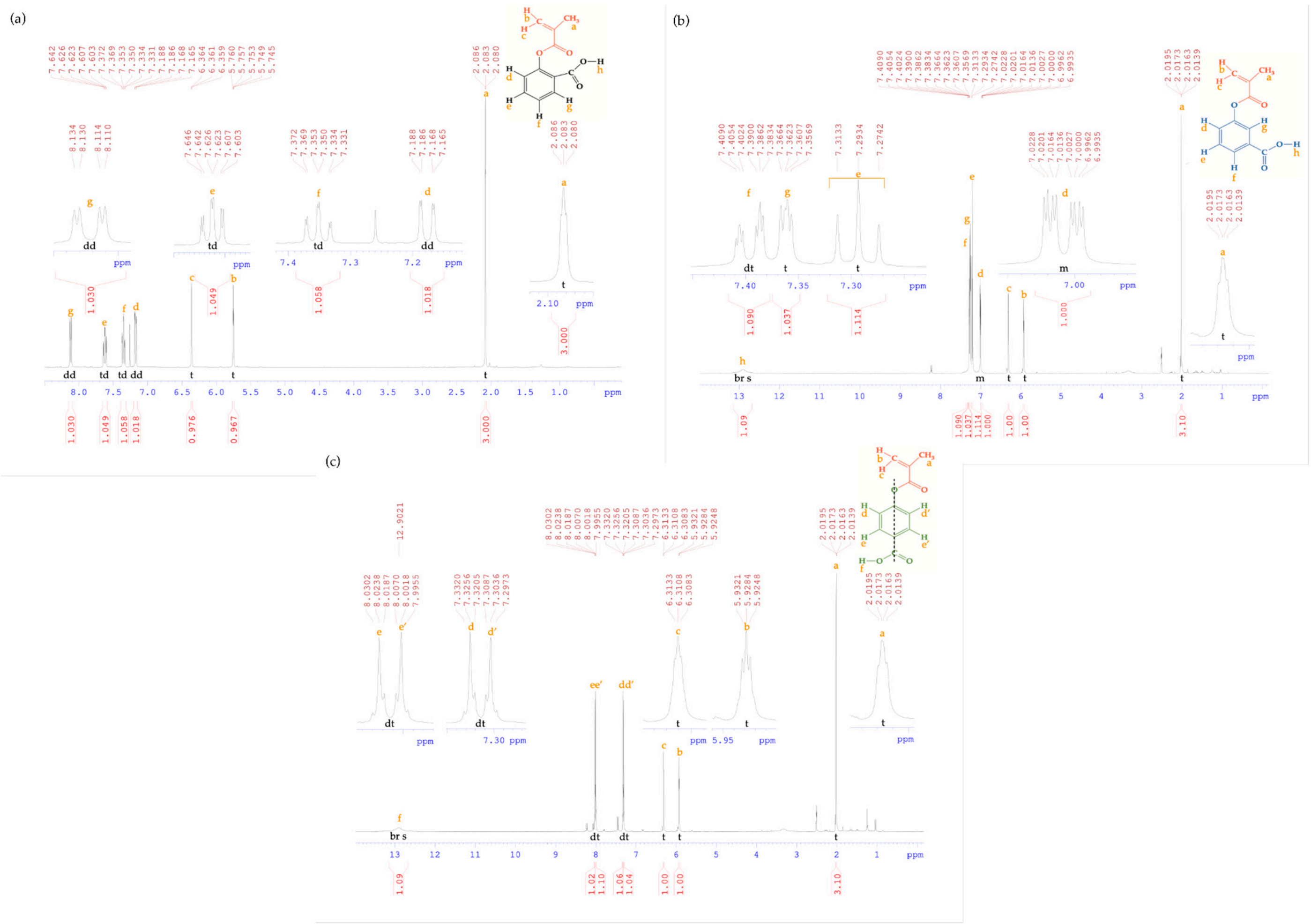

Figure S2. ^{1}H-NMR (400 MHz) spectra of **(a)** *o*-MBA, **(b)** *m*-MBA and **(c)** *p*-MBA dissolved in $CDCl_3$. The signals were assigned as follows: δ 12.9 (br s, 1H, carboxylic acid proton; observed for *m*-MBA and *p*-MBA); δ 8.2-7.0 (m, 4H, aromatic protons); δ 6.4-5.7 (two signals, 1H each, alkenyl protons); and δ 2.0 (t, 3H, methyl protons). MBA, methacryloyloxybenzoic acid; br s, broad singlet; s, singlet; d, doublet; dd, doublet of doublets; dt, doublet of triplets; td, triplet of doublets; t, triplet; m, multiplet.

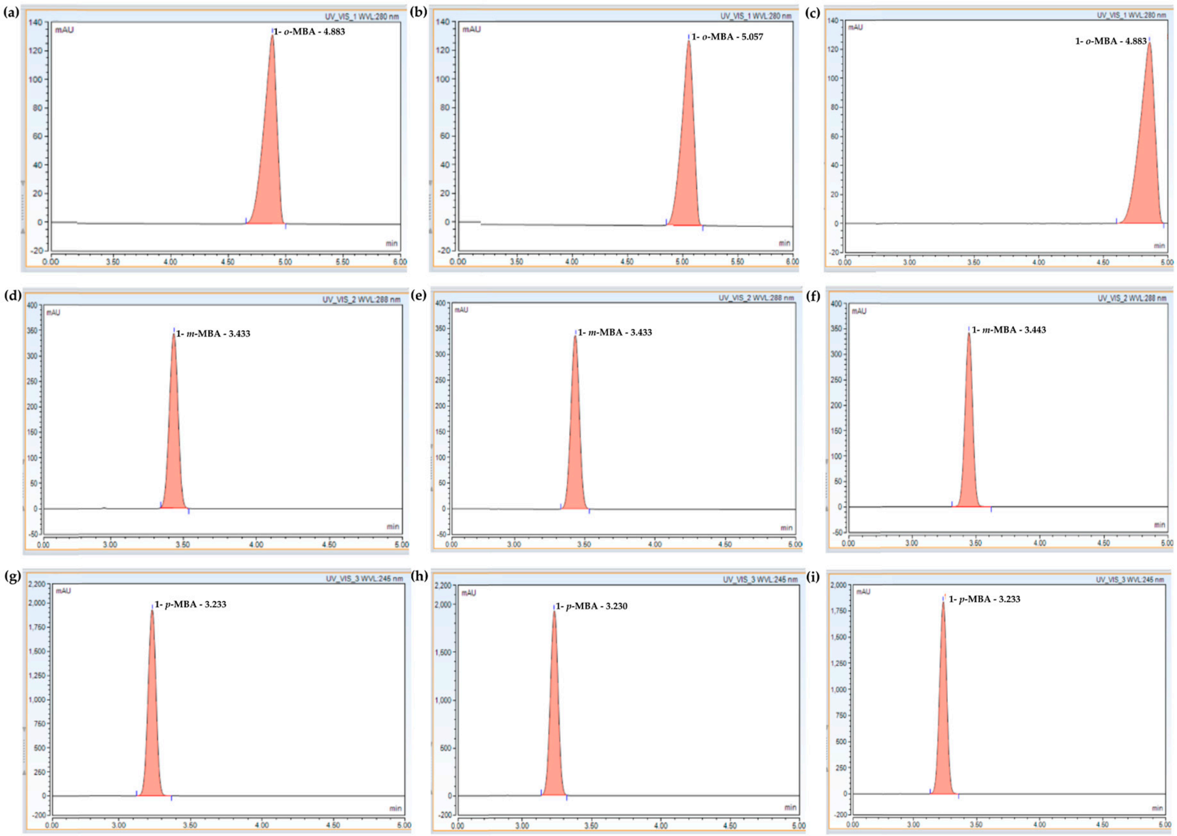

Figure S3. Representative HPLC chromatograms of standard solutions (200 µg/mL) of *o*-HBA, *m*-HBA, and *p*-HBA under simulated gastric (pH 1.2), intestinal (pH 6.8), and physiological (pH 7.4) conditions. Panels **(a–c)**, **(d–f)**, and **(g–i)** correspond to *o*-HBA, *m*-HBA, and *p*-HBA, respectively, while panels **(a, d, g)**, **(b, e, h)**, and **(c, f, i)** represent pH 1.2, 6.8, and 7.4 conditions, respectively. HBA: hydroxybenzoic acids.

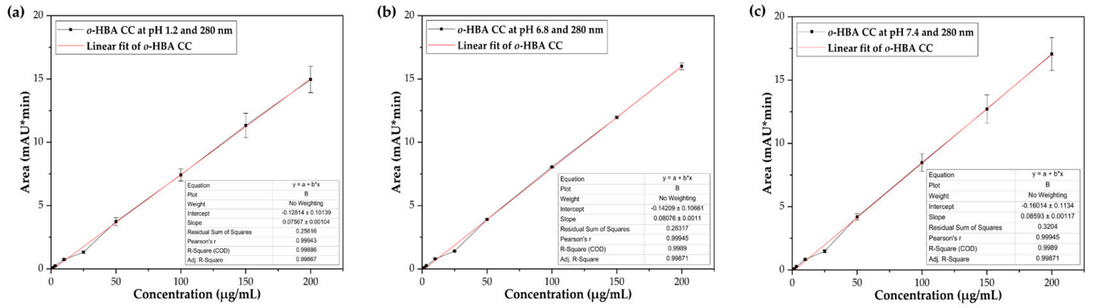

Figure S4. Calibration curves of *o*-HBA under simulated gastric (**a**, pH 1.2), intestinal (**b**, pH 6.8), and physiological (**c**, pH 7.4) conditions. Standard solutions were prepared under the same conditions as the analyzed samples. Data are presented as mean ± SD (n = 3), and linear regression analysis was performed for each pH condition. HBA: hydroxybenzoic acids.

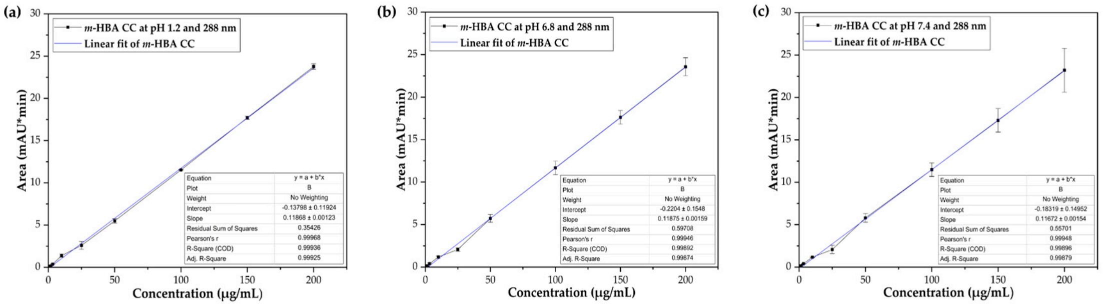

Figure S5. Calibration curves of *m*-HBA under simulated gastric (**a**, pH 1.2), intestinal (**b**, pH 6.8), and physiological (**c**, pH 7.4) conditions. Standard solutions were prepared under the same conditions as the analyzed samples. Data are presented as mean ± SD (n = 3), and linear regression analysis was performed for each pH condition. HBA: hydroxybenzoic acids.

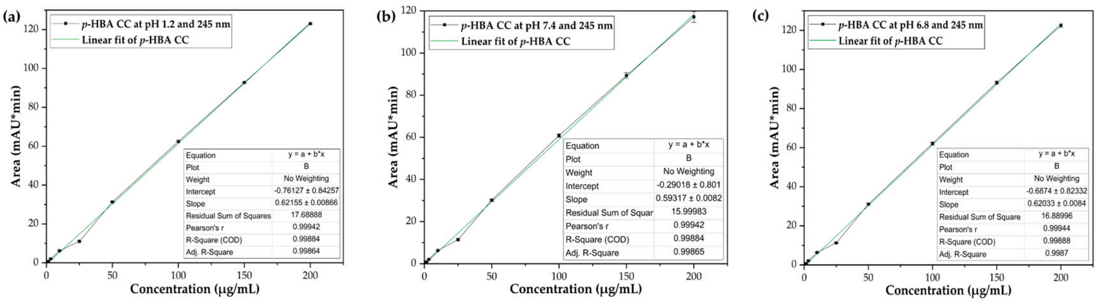

Figure S6. Calibration curves of *p*-HBA under simulated gastric (**a**, pH 1.2), intestinal (**b**, pH 6.8), and physiological (**c**, pH 7.4) conditions. Standard solutions were prepared under the same conditions as the analyzed samples. Data are presented as mean ± SD (n = 3), and linear regression analysis was performed for each pH condition. HBA: hydroxybenzoic acids.

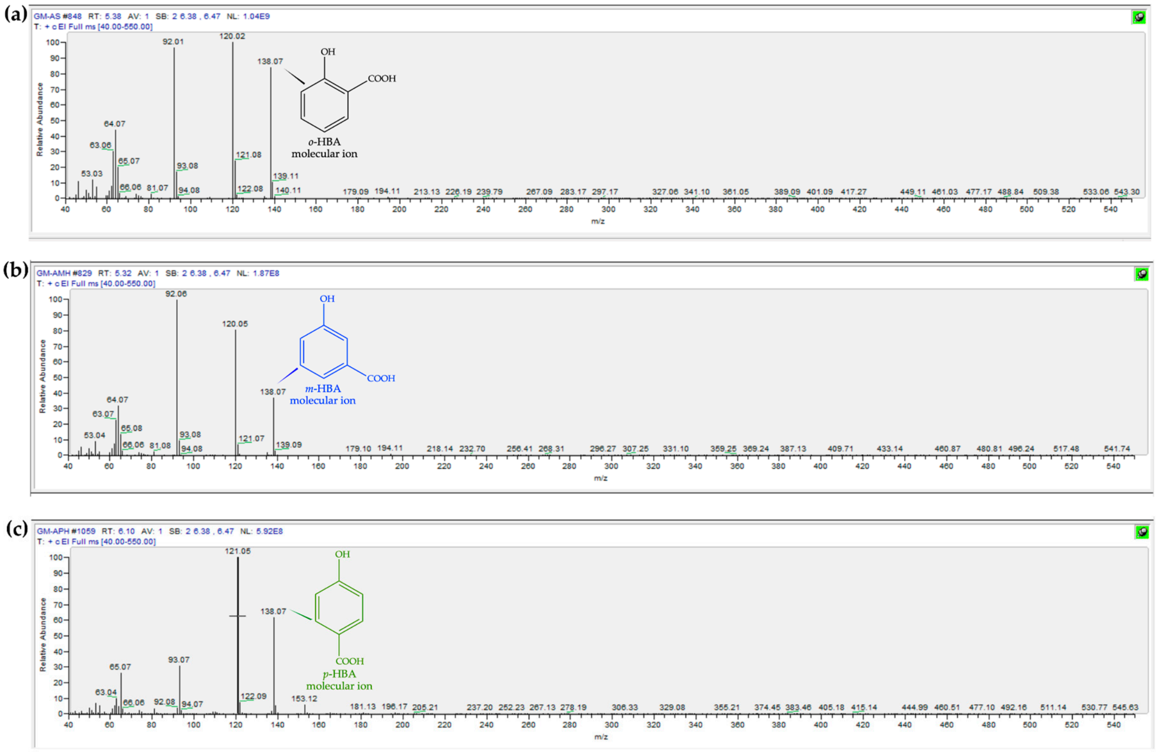

Figure S7. Mass spectra of **(a)** *o*-HBA, **(b)** *m*-HBA, and **(c)** *p*-HBA released from poly(*o*-MBA), poly(*m*-MBA), and poly(*p*-MBA), respectively, after 48 h of incubation at pH 7.4. HBA: hydroxybenzoic acids; MBA: methacryloyloxybenzoic acid.

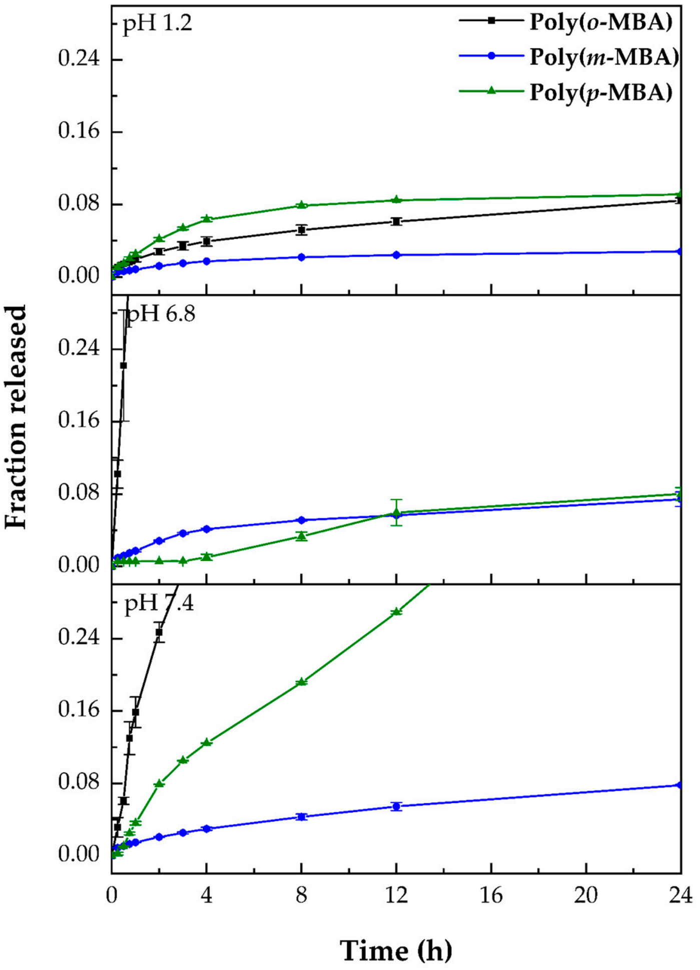

Figure S8. Cumulative fraction of HBA released from MBA polymer-based solid dosage forms as a function of time (0-24 h) at simulated physiological pH values of 1.2 (gastric fluid), 6.8 (intestinal fluid), and 7.4 (plasma). Release studies were conducted in aqueous buffer without organic solvents. Data are expressed as mean ± SD (n = 3). The y-axis was limited to a maximum released fraction of 0.25 to facilitate visualization of the early-stage release behavior. MBA: methacryloyloxybenzoic acid; HBA: hydroxybenzoic acid.

Table S1. Estimated kinetic parameters and goodness-of-fit and model selection criteria for Zero-order, First-order, Higuchi, and Korsmeyer–Peppas models applied to MBA polymer release profiles under different pH conditions.

Mathematical Model	Estimated Kinetic Parameters and Statistical Criteria for Model Fitting									
		Poly(*o*-MBA)			Poly(*m*-MBA)			Poly(*p*-MBA)		
		**pH 1.2**	**pH 6.8**	**pH 7.4**	**pH 1.2**	**pH 6.8**	**pH 7.4**	**pH 1.2**	**pH 6.8**	**pH 7.4**
Zero-order model	R^2_{adj}	0.57±0.1	0.83±9.4 ×10^{-2}	0.71±2.7 ×10^{-2}	0.47±9.8 ×10^{-2}	0.79±3.9 ×10^{-2}	0.45±0.1	-0.45±8.1 ×10^{-2}	0.78±8.9 ×10^{-2}	0.98±2.9 ×10^{-4}
	MSC	0.70±0.3	1.60±0.5	0.85±9.5 ×10^{-2}	0.37±0.2	1.27±0.2	0.33±0.2	-0.55±5.7 ×10^{-2}	1.43±0.4	3.90±1.7 ×10^{-2}

	AIC	44.3±3.2	50.4±2.8	29.8±0.4	-0.69±1.0	6.86±0.9	6.83±0.4	57.1±0.6	36.0±4.8	$38.8\pm5.8\times10^{-2}$
	k_0	$4.9\times10^{-3}\pm1.3\times10^{-4}$	$0.46\pm2.5\times10^{-3}$	$0.13\pm3.6\times10^{-3}$	$8.5\times10^{-3}\pm7.1\times10^{-5}$	$1.9\times10^{-2}\pm4.6\times10^{-4}$	$1.4\times10^{-2}\pm6.2\times10^{-4}$	$4.8\times10^{-3}\pm3.4\times10^{-5}$	$3.9\times10^{-3}\pm5.9\times10^{-4}$	$3.8\times10^{-2}\pm2.6\times10^{-4}$
First-order model	R^2_{adj}	0.60±0.1	$0.98\pm9.0\times10^{-3}$	$0.98\pm6.8\times10^{-3}$	-0.39±0.1	0.29±0.2	$0.84\pm1.1\times10^{-2}$	$-0.40\pm7.9\times10^{-2}$	$0.80\pm8.6\times10^{-2}$	$0.98\pm1.9\times10^{-4}$
	MSC	0.80±0.3	3.90±0.6	3.70±0.3	$-0.51\pm8.4\times10^{-2}$	0.19±0.3	$1.67\pm7.4\times10^{-2}$	-0.50±0.1	1.52±0.4	$4.13\pm1.5\times10^{-2}$
	AIC	43.4±3.3	34.1±5.2	43.1±2.8	29.1±0.1	45.4±1.0	35.0±0.7	56.6±0.6	35.1±4.9	36.5±0.2
	k_1	$5.3\times10^{-5}\pm1.6\times10^{-6}$	$9.8\times10^{-3}\pm6.5\times10^{-4}$	$1.9\times10^{-3}\pm5.6\times10^{-5}$	$1.5\times10^{-5}\pm3.2\times10^{-7}$	$4.4\times10^{-5}\pm4.6\times10^{-6}$	$5.2\times10^{-5}\pm3.3\times10^{-6}$	$5.2\times10^{-5}\pm4.1\times10^{-7}$	$4.2\times10^{-5}\pm6.6\times10^{-6}$	$4.9\times10^{-4}\pm4.4\times10^{-6}$
Higuchi model	R^2_{adj}	$0.99\pm1.5\times10^{-2}$	$0.79\pm9.3\times10^{-3}$	$0.94\pm6.5\times10^{-3}$	$0.73\pm3.5\times10^{-2}$	$0.92\pm4.8\times10^{-2}$	$0.98\pm5.1\times10^{-3}$	$0.65\pm3.2\times10^{-2}$	$0.87\pm1.7\times10^{-2}$	$0.87\pm6.0\times10^{-5}$
	MSC	5.12±1.8	$1.10\pm4.4\times10^{-2}$	2.60±0.1	1.15±0.1	2.58±0.8	4.00±0.3	$0.86\pm9.5\times10^{-2}$	1.94±0.1	$1.82\pm4.6\times10^{-4}$
	AIC	-4.2±19	21.1±1.3	40.6±1.1	10.8±0.6	19.1±6.6	9.52±4.8	41.5±1.0	30.4±4.1	59.6±0.1
	k_H	$0.22\pm8.3\times10^{-3}$	4.7±0.6	$2.33\pm7.1\times10^{-2}$	$7.5\times10^{-2}\pm1.3\times10^{-3}$	$0.19\pm1.5\times10^{-2}$	$0.21\pm1.2\times10^{-2}$	$0.24\pm2.9\times10^{-3}$	$0.17\pm2.4\times10^{-2}$	$1.09\pm7.5\times10^{-3}$
Korsmeyer–Peppas model	R^2_{adj}	$0.99\pm1.9\times10^{-3}$	$0.97\pm3.5\times10^{-2}$	$0.98\pm2.8\times10^{-3}$	$0.96\pm3.0\times10^{-3}$	$0.98\pm1.1\times10^{-2}$	$0.99\pm3.1\times10^{-3}$	$0.86\pm7.7\times10^{-3}$	$0.90\pm3.8\times10^{-2}$	$0.99\pm2.3\times10^{-4}$
	MSC	5.90±0.8	4.00±2.5	3.80±0.2	$2.90\pm7.0\times10^{-2}$	3.62±0.6	5.11±0.6	1.73±0.1	2.11±0.4	$4.62\pm3.1\times10^{-2}$
	AIC	-12.8±8	9.4±10.4	31.1±1.2	-8.27±1.4	7.7±4.45	-2.7±7.5	31.9±0.6	28.5±5.6	31.6±0.1
	k_{KP}	0.3±0.10	0.8±0.21	1.0±0.13	$0.28\pm1.5\times10^{-2}$	$0.42\pm7.4\times10^{-2}$	$0.12\pm3.9\times10^{-3}$	$0.96\pm6.0\times10^{-2}$	$6.8\times10^{-2}\pm2.5\times10^{-2}$	$9.9\times10^{-2}\pm3.3\times10^{-4}$
	n	$0.46\pm4.2\times10^{-2}$	$0.9\pm4.0\times10^{-2}$	$0.65\pm2.0\times10^{-2}$	$0.31\pm9.8\times10^{-3}$	$0.39\pm3.6\times10^{-2}$	$0.57\pm9.6\times10^{-3}$	$0.30\pm7.5\times10^{-3}$	$0.63\pm5.5\times10^{-2}$	$0.86\pm5.5\times10^{-4}$

Data are expressed as mean ± SD (n = 3). All parameter values and SD ≤ 10^{-2} were formatted in scientific notation to avoid precision loss from decimal rounding. *Abbreviations*. R^2_{adj}, adjusted coefficient of determination; MSC, model selection criterion; AIC, Akaike information criterion; k_0, Zero-order release constant (% · min^{-1}); k_1, First-order release constant (min^{-1}); k_H, Higuchi constant (% · $min^{-0.5}$); k_{KP}, Korsmeyer–Peppas release constant (% · min^{-n}); n, release exponent (used to infer the underlying drug-release mechanism); MBA: methacryloyloxybenzoic acid.

Table S2. Analytical validation parameters for the HPLC quantitative determination of HBA isomers (*o*-HBA, *m*-HBA, and *p*-HBA) across simulated dissolution media according to equations established in the Pharmacopoeia of the United Mexican States [1].

Isomer	Dissolution Medium	*S*	*b*	R^2	*RF*	*SD RF*	*%RSD*
o-HBA	pH 1.2	7.57×10^{-2}	-0.1281	0.9989	0.5504	1.04×10^{-2}	1.8882
	pH 6.8	8.59×10^{-2}	-0.1601	0.9989	0.5857	1.11×10^{-2}	1.8957

	pH 7.4	8.08×10^{-2}	-0.1421	0.9989	0.5886	1.14×10^{-2}	1.9406
	pH 1.2	0.1186	-0.1379	0.9994	0.9066	1.37×10^{-2}	1.5062
m-HBA	pH 6.8	0.1187	-0.2204	0.9989	0.8690	1.57×10^{-2}	1.8070
	pH 7.4	0.1167	-0.1831	0.9990	0.8644	1.45×10^{-2}	1.6823
	pH 1.2	0.6215	-0.7612	0.9988	4.6110	8.12×10^{-2}	1.7618
p-HBA	pH 6.8	0.6203	-0.6874	0.9989	4.6669	7.57×10^{-2}	1.6210
	pH 7.4	0.5931	-0.2901	0.9988	4.5912	6.03×10^{-2}	1.3138

Experimental conditions: Matrix-matched calibration curves were constructed across a linear range of 8 points between 1.5 - 200.0 μg/mL in simulated gastric fluid (pH 1.2), simulated intestinal fluid (pH 6.8), and simulated physiological fluid (pH 7.4). UV detection wavelengths: λ = 280 nm for *o*-HBA, λ = 288 nm for *m*-HBA, and λ = 245 nm for *p*-HBA. *Abbreviations*. *S*: slope of the matrix-matched calibration curve (mAU·min·mL/μg); *b*: *y*-intercept of the calibration curve (mAU·min); R^2: coefficient of determination derived from linear least-squares regression (n = 3 independent runs); *RF*, Response Factor (mAU·min·mL/μg); *SD RF,* Standard Deviation of RF (mAU·min·mL/μg); %*RSD*, Relative Standard Deviation; HBA: hydroxybenzoic acids.

References

1. Secretaría de Salud; Comisión Permanente de la Farmacopea de los Estados Unidos Mexicanos. *Pharmacopoeia of the United Mexican States*, 12th ed.; Secretaría de Salud: Mexico City, Mexico, 2018.

Disclaimer/Publisher's Note: The statements, opinions and data contained in all publications are solely those of the individual author(s) and contributor(s) and not of MDPI and/or the editor(s). MDPI and/or the editor(s) disclaim responsibility for any injury to people or property resulting from any ideas, methods, instructions or products referred to in the content.
